# Canine hyper-sociability structural variants associated with altered three-dimensional chromatin state

**DOI:** 10.1186/s12864-024-10614-6

**Published:** 2024-08-07

**Authors:** Dhriti Tandon, Enikő Kubinyi, Sára Sándor, Hannah Faughnan, Ádám Miklósi, Bridgett M. vonHoldt

**Affiliations:** 1 Department of Ecology and Evolutionary Biology, Princeton University, Princeton, NJ USA; 2 Department of Ethology, ELTE Eötvös Loránd University, Budapest, Hungary; 3 MTA-ELTE Lendület “Momentum” Companion Animal Research Group, Budapest, Hungary; 4 ELTE NAP Canine Brain Research Group, Budapest, Hungary

**Keywords:** Chromatin, Hyper-sociability, Domestication, Transposable elements

## Abstract

Strong selection on complex traits can lead to skewed trait means and reduced trait variability in populations. An example of this phenomenon can be evidenced in allele frequency changes and skewed trait distributions driven by persistent human-directed selective pressures in domesticated species. Dog domestication is linked to several genomic variants; however, the functional impacts of these variants may not always be straightforward when found in non-coding regions of the genome. Four polymorphic transposable elements (TE) found within non-coding sites along a 5 Mb region on canine CFA6 have evolved due to directional selection associated with heightened human-directed hyper-sociability in domesticated dogs. We found that the polymorphic TE in intron 17 of the canine *GTF2I* gene, which was previously reported to be negatively correlated with canid human-directed hyper-sociability, is associated with altered chromatin looping and hence distinct *cis*-regulatory landscapes. We reported supporting evidence of an *E2F1*-DNA binding peak concordant with the altered loop and higher expression of *GTF2I* exon 18, indicative of alternative splicing. Globally, we discovered differences in pathways regulating the extra-cellular matrix with respect to TE copy number. Overall, we reported evidence suggesting an intriguing molecular convergence between the emergence of hypersocial behaviors in dogs and the same genes that, when hemizygous, produce human Williams Beuren Syndrome characterized by cranio-facial defects and heightened social behaviors. Our results additionally emphasize the often-overlooked potential role of chromatin architecture in social evolution.

**Supplementary Information:**

The online version contains supplementary material available at 10.1186/s12864-024-10614-6.

## Background

Social behavior is a quantitative complex trait defined through numerous biological, life history, developmental, and cognitive profiles. While candidate loci methods have identified genes encoding hormones, sensory receptors, or neurotransmitters that shape behaviors [1, 2], pleiotropy and polygenic infrastructure reduce the clarity of these primary structural findings [3]. The addition of multi-*omic* data to candidate loci findings has now uncovered large-effect variants that act as master regulatory loci shaping behavior, by rapidly innovating pleiotropic effects on transcription, chromatin conformations, and the underlying biological pathways [4–6]. More recently, genomic architecture has been implicated in the evolution of social behavior due to mechanisms of tightly linked supergene complexes [7, 8], copy number of structural variants (SVs) [9, 10] and, to a lesser extent, transposable elements (TEs) [11, 12]. TEs have the propensity to alter cis- regulation via coordinated genetic and epigenetic mechanisms. Such elements are targeted by the genome surveillance machinery for TE inactivation through various mechanisms, including DNA methylation and histone modifications [13, 14], with the epigenetically silenced TEs exerting cis-regulatory impacts on proximal (i.e., within 100 nucleotides) transcriptionally active sequences [15, 16]. TEs also harbor binding sites for transcription factors and regulatory proteins that impact higher order three-dimensional (3D) chromatin structure [17], making them strong candidates for drivers of phenotypic change and even social evolution.

Species domestication results from the rapid and significant phenotypic evolution in response to novel human-directed selective pressures, exemplified in the Farm-fox experiment where behavioral and morphologic evolution was documented [18]. Dogs were the first domesticated species [19], where strong artificial selection maintains strict impermeable boundaries between dog breeds and has removed a significant amount of intra-breed genomic and phenotypic variation, enhancing the efficiency of mapping and evolutionary studies [20, 21]. Among the several well-known morphological and behavioral changes that occurred during the domestication of gray wolves to dogs [22–24], human-directed sociability is one of the prominent phenotypes suspected to have been selected during domestication [25].

Dogs exhibit a magnified social interest towards humans, referred to as hyper-sociability [26, 27]. Through strong directed selection, this social phenotype has provided feedback to the underlying molecular infrastructure, evidenced by moderate estimates of heritability (h^2^, social interactions towards humans = 0.23) with notable differences across breeds (h^2^, willingness to make contact with humans: German shepherds = 0.38, Rottweilers = 0.03) [28]. Part of the supportive molecular infrastructure includes four previously mapped large-effect polymorphic retro-transposable elemental (TE) sequences, found on canine chromosome CFA6 with copy number of the derived allele positively correlated with human-directed hyper-sociability [27]. The derived alleles are located within or proximal to three genes: two TE insertions in intron 1 of Williams-Beuren Syndrome Chromosome Region 17 (*WBSCR17*), the lack of an insertion in intron 17 of the General Transcription Factor 2-I (*GTF2I*), and one insertion in intron 5 of *POM121* Transmembrane Nucleoporin (*POM121*) [27].

Here, we quantify the functional impacts of the TE sequence polymorphism, specifically the presence of a tRNA-based *S*hort-*I*nterspersed *N*ucleotide *E*lement in intron 17 of canine *GTF2I* [27] that encodes a multi-functional and ubiquitously expressed transcription factor 2-I (TFII-I) protein [6]. Within dogs, vonHoldt et al. (2017) [27] reported that the allele containing the TE insertion in gene *GTF2I* was the minor allele (*f* = 0.31 or 5/16 dogs). Concordant with the neural crest cell hypothesis [29], *GTF2I* is a plausible master regulator for domestication-driven morphological and behavioral phenotypes due to its involvement in neural tube closure and neural crest cell migrations [30]. Throughout embryogenesis, *GTF2I* is necessary for neuronal development [30] and is later central in regulating synaptic plasticity and biochemical pathways for anxiety and sociability [6, 31]. Perhaps more striking is that artificially constructed hemizygous knockouts of *GTF2I* are associated with heightened sociability in murine systems [32] and a large-scale naturally occurring hemizygous deletion on human chromosome 7, which includes *GTF2I*, causes Williams Beuren Syndrome (WS) [33], a neurodevelopmental disorder that is characterized by cranio-facial defects and extremely high levels of social behaviors [34, 35].

Although the TE found within *GTF2I*’s intron is strongly associated with the evolution of hyper-sociability in domestic dogs, the proximate mechanisms are currently undescribed. This is especially compounded by the fact that the high-effect TE is located within a non-coding region, 700 nucleotides from the nearest exon, and is relatively small (only 187 bp) as compared to most high-impact structural variants located in intronic and intergenic regions [27]. With past evidence for associative altered *cis*- regulation in canine blood [36], we hypothesize that the copy number of this intronic TE shifts the 3D chromatin state and the *cis*- regulatory landscape, which further impacts downstream transcriptional activities. We investigated chromatin architecture and gene expression differences associated with *GTF2I*’s intronic TE in dog brainstems, a tissue relevant to social behaviors as it contains neuronal projections for glutamatergic, serotonergic and dopaminergic neurons [37]. We found that the polymorphic TE located in intron 17 of *GTF2I* is associated with altered chromatin looping with its own intron 1 and putative alternative splicing of this gene. At a more global level, we find differences in pathways related to the extra-cellular matrix associated with the ancestral and derived forms of this gene.

## Results

### Ancestral TE insertion associated with higher expression of GTF2I exon 18

**Fig. 1:**
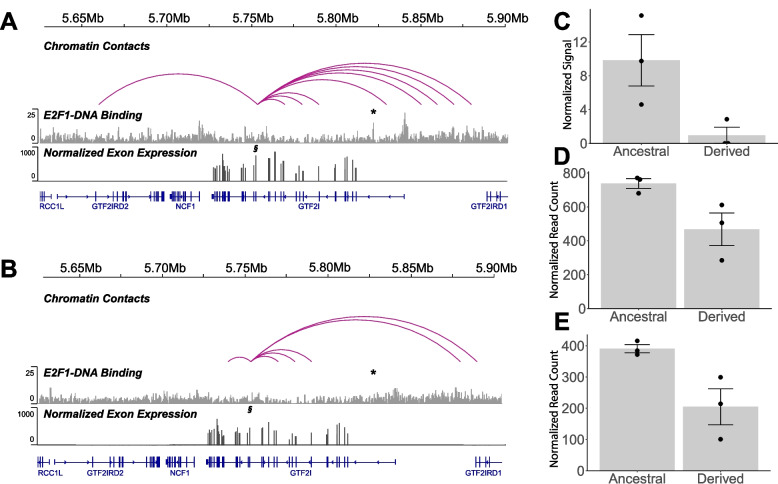
Differences in *cis-* regulation of *GTF2I* exon 18, between ancestral TE present and derived TE absent states of *GTF2I*. Visualization of the target region with chromosomal coordinates in Mb (top line) along canine chromosome CFA6 for *Capture C* contacts (top), average *E2F1 ChIP-Seq* Coverage (middle) with differential peak (*), and average normalized exon expression with exon 18 (§) values at *GTF2I* (bottom) for samples **A** heterozygous for the ancestral TE insertion in *GTF2I* and **B** homozygous for the derived allele lacking the TE insertion in *GTF2I*. For panels **C-E**, the ancestral state refers to the TE insertion in *GTF2I,* while the derived allele is the lack of the TE insertion. **C** Normalized *ChIP-Seq* signal for *E2F1* located at intron 1 of *GTF2I* (chr6:5,822,073–5,822,473 bp, *p*_*adj*_ = 0.03). **D** Expression of *GTF2I* exon 18 (*p*_*adj*_ = 0.138) as reported in the IGV track. **E** *GTF2I* exon-17—exon18 junction expression (*p*_*adj*_ = 0.141). Black circles show each data point, bar heights correspond to group means, and error bars correspond to standard errors

We collected data from brainstem tissue (pons) of six male dogs (12–16 years old) from three genomic dimensions: targeted chromatin conformation capture sequencing (*Capture C*) at the polymorphic TE site, *E2F1* and *H3K27ac* chromatin immunoprecipitation sequencing (*ChIP-seq*), and RNA sequencing (*RNA-Seq*). Three of the six dogs were heterozygous for the ancestral allele, which contains the TE insertion, while the other three dogs were homozygous for the derived allele that lacked a TE insertion [27] (Table S1). To reduce the likelihood of false positives, we only considered 3D contacts with a concordant differential *ChIP* peak signal as biologically meaningful. We found that the TE sequence itself was enriched for binding motifs for the transcription factor *E2F1* and its co-factors (see Supplementary Text). In humans, *E2F1* has binding sites at genes within 1 Mb upstream and downstream (i.e. in *cis*- conformations) of *GTF2I* (see Supplementary Text). Samples that carried at least one copy of the ancestral allele exhibited unique 3D contacts, which we quantified from the contact frequency at each site relative to the surrounding background [38], with three regions on CFA6: intron 1 of *GTF2IRD2* at 5.65–5.67 Mb, intron 1 of *GTF2I* at 5.82–5.84 Mb, and upstream of *GTF2I* spanning 5.85–5.87 Mb (log *q* < -10) (Fig. 1A; Table S2). When samples carried the derived allele lacking the TE, we discovered unique contacts with a novel CFA6 bin at 5.73–5.75 Mb (log *q* < -10) (Fig. 1B; Table S2). We conducted a negative binomial Wald Test in the R package *DiffBind*, to investigate differences in *ChIP* peak enrichment between samples as a function of mean signal quantified by normalized read counts in each peak bin [39]. Hence, *ChIP* peaks with significant differences, hereafter referred to as “differentially enriched peaks”, are those whose signal strength differs more between samples with different genotypes than among samples with the same genotype [39]. We found significant differences in the *ChIP* peak signal for *E2F1* at CFA6:5,822,073–5,822,473 within intron 1 of *GTF2I* (log_2_ Fold Change _[Derived/Ancestral]_ = -3.12; *p* = 6.73 × 10^–4^; *p*_*adj*_ = 0.03), which is located within the TE containing contact site at 5.82–5.84 Mb (Figs. 1A,C). We analyzed exon expression of the six tissues which were controlled for age and sex, to account for any differences that are developmental-stage dependent expression patterns for *GTF2I* isoforms [40, 41] (see Supplementary Text). We used an R package *JunctionSeq* to conduct a likelihood ratio test to determine the effects of TE genotypic state on exon or splice junction expression [42]. We quantified exon expression using reads mapped within the exonic bins and splice junction expression using reads mapped across two exons [43], such that these reads visually mimic a discordant alignment similar to a deletion representing the spliced-out intron. We found higher usage of exon 18 (log2 Fold Change_[Derived/Ancestral]_ = -0.528, *p* = 0.001, *p*_*adj*_ = 0.138) and evidence for a splice junction between exon 17 and 18 (log2 Fold Change_[Derived/Ancestral]_ = -0.783, *p* = 0.001, *p*_*adj*_ = 0.141) as a function of the TE insertion state at *GTF2I* (Figs. 1D,E; Table S3). There were no changes in expression levels of *GTF2I* itself with respect to the TE insertion (*p* > 0.1). We found weak evidence for an additional loop between the polymorphic TE site and the gene *LIMK1* (Fig. S1; see Supplementary Text).

### Differences in the enrichment of biological pathways related to extra-cellular matrix

**Fig. 2:**
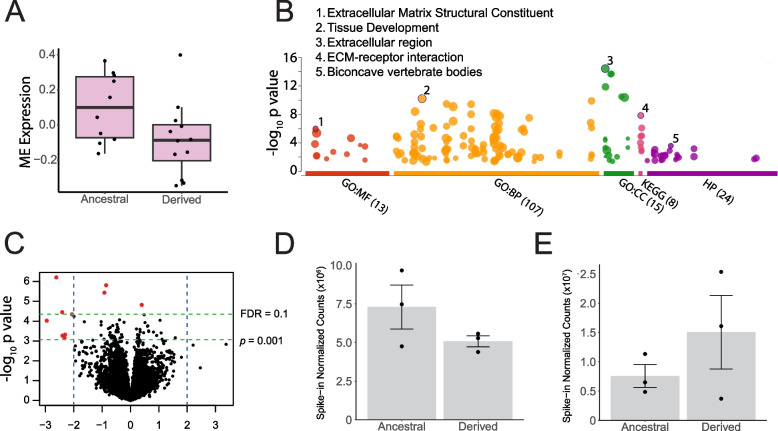
Downstream molecular impacts on global gene expression and regulation. **A** Box-and-whisker plot of module eigengene (ME) expression for the differentially expressed module. ME expression values have significantly different means (*t* = 2.1348, *df* = 19.744, *p* = 0.0455) between samples containing the ancestral and derived TE genotypes at GTF2I. Whiskers represent the data range excluding outliers. Horizontal edges correspond to 25th, 50th and 75th percentiles. **B** Gene ontology (GO) hits for genes in the differentially expressed module displaying their -log_10_*p* for each GO categories: Molecular Function (MF), Biological Processes (BP), Cellular Component (CC), Kyoto Encyclopedia of Genes and Genomes pathways (KEGG) and Human Phenotype (HP). Top GO term in each category is numbered and keyed. **C** Volcano plot of differentially expressed genes (red points; FDR < 0.1, or *p* < 0.001 and log_2_ fold change >|2|), for the 22 samples. **D** Spiked-in normalized read counts for *E2F1* enrichment and; **E** *H3K27ac* marks. Bar heights represent mean values, error bars correspond to standard errors, and black circles depict replicate values

Since *GTF2I* encodes a transcription factor regulating the expression of several genes [6], we evaluated the potential downstream impacts on global biological pathways as a function of TE genotypic state at *GTF2I*. First, we looked for differentially expressed genes across genome and found 10 genes differentially expressed as a function of the TE copy number at *GTF2I* (Fig. 2C; Table 1). Next, we constructed gene modules, which are sets of co-expressed genes that broadly represent biological pathways [44]. Since the construction of gene modules requires at least 12 samples [44], we collected *RNAseq* data from the brainstem tissue of 16 additional dogs across ages, sexes and breeds (Table S1) and conducted these analyses on all 22 samples with *RNAseq* data, while accounting for confounding variables (*Supplementary Information: Materials and Methods*). We generated signed gene networks and quantified module eigengene (ME) values, to represent the dimensionality reduced (i.e. the first principal component) gene expression values from all genes in the module, which are broadly associated with changes in biological pathways [44]. Since signed modules contain co-expressed genes with the same directionality of expression change (i.e., all with increased or decreased expression for a given condition) [44], these were preferred over the generation of unsigned modules to ease the interpretation of results and identify biological pathways with a general increased or decreased enrichment in the derived state of *GTF2I*. We found a single gene module with higher expression for samples carrying the ancestral *GTF2I* TE insertion (Pearson Correlation coeff _[Derived]_ = -0.43; *p* = 0.047; p_adj_ = 0.63); however, this did not survive multiple comparison corrections (Figs. 3A, S2A-D; Table S4). Since this was the only gene module meeting the significance threshold of *p* < 0.05, we hereafter refer to this module as the “differentially expressed module”. We then conducted a Gene Ontology Enrichment analysis on g*:Profiler* [45] using IDs of genes within the differentially expressed gene module. The top ranked gene ontology term is for the cellular component “extra-cellular region” (*p*_*adj*_ = 8.54 × 10^–14^), and the top KEGG pathway [46] is for “Extra-Cellular Matrix (ECM) receptor interaction” (*p*_*adj*_ = 3.81 × 10^–8^) (Fig. 2B). We further found *BNC2* among the top ranked transcription factors candidates that putatively regulates genes in the differentially expressed gene module (Mean Rank = 10.33; Percentage overla*p* = 28.6%; *Enrichr *[47–49] FDR = 5.68 × 10^–18^; *GTEx *[50] FDR = 2.51 × 10^–5^; *ARCHS4* [51] FDR = 8.70 × 10^–17^). *BNC2* was also found to be a differentially expressed gene (Tables 1, S5).

**Table 1:** Significantly differentially expressed genes with their respective false discovery rate (FDR < 0.1), log2 fold change ratios (log2FC > 2), and significance* p* values (*p* < 0.001)

Gene Symbol	Log2FC	*p* value	FDR
*SLC47A1*	-2.615	6.19 × 10^–7^	0.010
*SLC14A1*	-0.857	1.53 × 10^–6^	0.012
*BNC2*	-0.919	3.67 × 10^–6^	0.019
*PPIP5K1*	0.397	1.51 × 10^–5^	0.059
*ERMAP*	-2.064	4.53 × 10^–5^	0.107
*ADAMTS15*	-2.409	3.53 × 10^–5^	0.107
*SLC6A20*	-2.953	9.43 × 10^–5^	0.123
*WNT16*	-2.399	5.38 × 10^–4^	0.183
*CCN3*	-2.307	4.80 × 10^–4^	0.183
*SLC22A6*	-2.327	6.99 × 10^–4^	0.193

Given *GTF2I*’s multi-functional role in transcriptional and translational regulation [6, 52, 53], we additionally identified binding motifs at differentially enriched *E2F1* and *H3K27ac* peaks (as identified by *DiffBind analyses*) between samples with differential *GTF2I* genotypes. We conducted the motif enrichment analysis using the web-based tool, *CentriMo* [54], against the JASPAR2022 CORE vertebrates non-redundant v2 database [55]. We did not find enriched binding motifs among regions harboring differential histone acetylation marks. Differentially enriched *E2F1* peaks with higher signal in samples with the derived allele (log2FC > 2; FDR < 0.1) were significantly enriched for binding motifs of *ZNF740* (e = 6.6 × 10^–6^ to 9.1 × 10^–3^; percent matching = 70.0%). However, differentially enriched *E2F1* peaks with higher signal in samples with the ancestral allele (log2FC > 2; FDR < 0.1) did not present any significantly enriched motifs. We found no changes in overall *H3K27ac* and *E2F1* enrichment (*p* > 0.1) (Figs. 2D, E).

## Discussion

### Linking Chromatin Architecture to Behavioral Evolution

Functional changes driven by intronic TEs have been well studied, although typically, such TEs are large in size and physically proximal to the nearest exon (i.e., within 100 nucleotides) or splice sites [56–58]. Our work confirms that the polymorphic 187 bp intronic TE alters *cis*-regulatory landscapes within CFA6:5.7–6.3 Mb, by impacting the chromatin structure itself. The TE promotes altered looping with intron 1 of *GTF2I*, which are further associated with differences in gene regulation. To our knowledge, the study provides the first evidence for a gene loop that is associated with social evolution as a consequence of animal domestication.

Our proposed model for the altered loop state is that the TE offers a binding site for an *E2F1* co-factor with subsequent recruitment of the *E2F1* transcription factor itself, which binds to an intron 1 site of *GTF2I*. This would facilitate looping between the TE insertion and chr6:5.82–5.84 Mb. The loop and its concordant *E2F1* peak are lost when *GTF2I* does not contain the TE in the derived state, which could be a consequence of binding site loss for the *E2F1* co-factor and the lack of *E2F1* in *GTF2I* intron 1. Given the proposed model, we would anticipate a peak in intron 17 of GTF2I. However, we could not survey DNA–protein binding activity at the TE itself due to its repetitive nature. We instead relied on chromatin conformation evidence that emerged from the polymorphic TE and a concordant *E2F1* peak at the putative contact site. In addition, our *in-silico* discovery also suggested that a co-factor of *E2F1*, such as *Sp1* (see Supplementary Text), rather than *E2F1* itself, could bind to the polymorphic TE. Hence, a lack of enrichment of this region could also be explained by immunoprecipitation targeting *E2F1* and compromised protein–protein interactions during the *ChIP* prep. Strikingly, we find no changes in expression levels of *GTF2I* itself; rather, we find an associated change in a single exon of *GTF2I* after controlling for confounding variables. While the differential exon usage analysis marginally misses the FDR < 10% threshold, multiple comparison corrections were performed with respect to all exons in the genome, including those of non-coding RNAs. Given this large sample size of exons, we still indicate that these results have the potential to be biologically meaningful and worthy of follow-ups for isoform discovery through long-read sequencing methods such as Iso-Seq. While we have not identified a causal mechanism linking the *E2F1* loop and splicing, *E2F1* is known to interact with splice-impacting co-factors such as the p100/TSN complex and regulates the splicing patterns of its target genes [59]. An alternative mechanism is that the altered looped state itself, rather than the molecular functions of *E2F1*, contributes to splicing that is facilitated by alternate promoter adoption whereby different promoters could express different isoforms of the same gene [60, 61].

### Potential Convergence in Gene Regulatory Mechanisms Underlying Hypersocial Behavior

*GTF2I* exon 18 in humans encodes the R3 domain of the TFII-I protein [52, 62], which consists of a DNA binding leucine zipper domain followed by six loop-helix-loop repeat domains (R1-R6). The TFII-I loop-helix-loop domains are involved in protein–protein interactions [53, 62] and changes to the R3 domain could hence alter protein interactions with TFII-I. Although we did not directly investigate the changes in protein interactions with TFII-I to establish causality, our correlative evidence suggests the convergent outcome of reduced expression of *BNC2* and reduced expression of a gene module that includes *BNC2* target genes in samples that lack the TE insertion in *GTF2I*. Among the transcription factors that target *BNC2* include *USF1* and *MYC*, which have incidentally been identified as protein interactors of TFII-I [53, 62, 63]. Altered *GTF2I* exon 18 expression could drive changes in TFII-I proteomic interactions with *USF1* and *MYC*, thereby impacting molecular processes affecting canine hypersocial behavior. TFII-I also interacts with the histone deacetylase 3 protein (HDAC3) [64]. This protein facilities the removal of histone acetylation marks, thereby acting as a gene silencer in most cellular contexts [65]. While our findings suggest that samples with the ancestral allele of the TE insertion carry lower levels of global *H3K27ac*, this difference was not significant. This could be due to a variety of reasons that include an underpowered design for the specific analyses type, combinatorial confounding impacts of other histone deacetylases and nuanced proteomic impacts on TFII-I such that interactions with some proteins, and not others, are affected. Hence, future efforts to determine interactomes of TFII-I in samples with ancestral and derived forms of *GTF2I* could help identify downstream biological impacts of the TE locus. However, these assays are currently technically challenging due to a limited availability of fresh tissues or cell lines from dogs.

In addition to higher sociability, patients diagnosed with Williams Beuren Syndrome also have cranio-facial abnormalities, explained by extra-cellular matrix anomalies [34]. Our results pertaining to gene module differences suggest that biological pathways related to the extra-cellular matrix show reduced expression. We also see functional changes related to the gene elastin (*ELN*), which is included in the differentially expressed gene module. Patients with WS can have varying lengths of deletions at the 7q11.23 locus, and 90% of patients with WS have a hemizygous deletion of *ELN* [66]. Extra-cellular matrix anomalies may not directly explain neurocognitive profiles and hence social behaviors. However, our results suggest that the derived *GTF2I* allele recapitulates some molecular characteristics of WS in domestic dogs: altered expression of extra-cellular matrix-related pathways and variants impacting *GTF2I* function.

## Conclusion

While often in an “arms-race” with the host and hence usually silenced, few TEs can be co-opted into regulatory sequences that promote specific transcriptional modules. Regulatory DNA sequences usually undergo purifying selection when the selective environment is stable [67]. However, new selective pressures that favor novel phenotypes will drive directional selection on regulatory loci. Such is the case for the canine intronic TE at *GTF2I*, which shows distinctive allele frequency differences between the ancestral gray wolf and dog genomes [27], with the wolf genome ‘co-opting’ the TE and the dog genome ‘purging’ it. While showing clear signatures of selection, functional impacts of TEs outside of coding regions are currently understudied across evolutionary scenarios, with most investigations limited to first-order genomic structure, overall TE distribution across the genome, and their putative relationship to candidate loci [12]. While more work is warranted to prove causality, we emphasize the relevance of high-effect non-coding variants and their role in regulating complex phenotypes in non-model systems by reporting their potential and indirect impacts on gene regulation through altered chromatin states. While our study does not prove this, we provide an evolutionary interpretation where the non-looped state of *GTF2I* could possibly provide a dog-specific fitness advantage from increased human-directed sociability. Molecular changes associated with an altered regulatory landscape can also introduce fitness costs [68] due to a loss in existing transcriptional machinery that could impact the extra-cellular matrix and activity of the multi-functional *GTF2I* gene, which could be validated by future efforts. We hope to provide a new framework for exploring the molecular infrastructure of social evolution by viewing the genome as more than a culmination of base-pairs and combining higher-order information on 3D genome architecture.

### Limitations

Due to a lack of ability to do functional experiments and CRISPR-based gene editing in dogs, the polymorphic TE locus within *GTF2I* is only correlatively linked to hyper-social behaviors. In addition, the bulk of our findings that pertain to *cis*-regulation have been conducted in male dogs (12–16 years old). This represents technical difficulties with sample acquisition from dog brains as most euthanized younger dogs have serious health complications and hence may present results confounded by these complications. Upon limiting samples to those with no obvious brain-related conditions, we were unable to obtain a sample size powerful enough to reliably conduct this study on only early-life or female dogs. Therefore, we caution readers that some specificities of gene regulation may be age or sex specific. Owing to low cross-correlation scores of our E2F1 *ChIP* data, some E2F1-DNA binding sites may be missed. We also caution that results pertaining to differential gene module analysis did not survive FDR corrections, owing to the presence of a single gene module passing a significance threshold of *p* < 0.05. Nonetheless, we present substantial evidence supporting the propensity of this TE to impact *cis*-regulation through an altered chromatin state.

## Methods

We obtained pons brainstem tissues preserved in *RNALater* (Thermo Fisher Scientific, Waltham, MA, USA), for 22 dogs collected by the Canine Brain and Tissue Bank (CBTB) at Eötvös Loránd University, Budapest, Hungary. Each sample was associated with metadata that included age, sex, and breed information (pure or mixed) (Table S1). Six of the 22 dog samples had an additional paired specimen available, which had been flash frozen at the time of collection. For these flash-frozen samples, we carried out *Capture C*, targeted at the polymorphic *GTF2I* TE site (CanFam 3.1; CFA6:5,753,797–5,753,983), and *ChIP-Seq* to quantify E2F1-DNA binding peaks and H3K27ac regions at chromatin loop bases. We collected RNA-seq data to further investigate differences in local gene regulation associated with the chromatin loops for the six samples. We conducted a higher-powered analysis of global differences in biological pathways among all 22 dog brainstem samples. Detailed methods, protocols and software used can be found in *Supplementary Information: Materials and Methods*.

### Supplementary Information

Supplementary Material 1.

## Data Availability

Aligned and sorted BAM files are available through the Short Read Sequencing Archive (ncbi.nlm.nih.gov/sra; Bioproject number: PRJNA939639). Raw and processed ChIP-Seq data is additionally available through Gene Expression Omnibus (GSE232642). Sample metadata is available in Table S1, and library statistics are available in Table S7-S9.
