# Supplementary material for: Canine hyper-sociability structural variants associated with altered three-dimensional chromatin state
Source: BMC Genomics. 2024 Aug 7;25:767. doi: 10.1186/s12864-024-10614-6 (PMC11305043; doi:10.1186/s12864-024-10614-6)
Supplement: Supplementary file 1 — Supplementary Material 1. [file 12864_2024_10614_MOESM1_ESM.docx]

**Supplementary Information**

Materials and Methods

Supplementary Text

Fig. S1. Differences in *cis-* regulation of *LAT2*, between ancestral TE present and derived TE absent states of *GTF2I*

Fig. S2. Construction of gene modules on WGCNA.

Fig. S3. Multiple comparisons adjustment for conservative *p*-values distributions

Fig. S4. *ChIP-Seq* binding profile for E2F1 and H3K27ac.Fig. S5. Principal component loadings of all 22 samples based on RNA-Seq count values.

Table S1. Sample Metadata with Sample ID, genotypic state at WBSCR17 site 1 (cha6.6), genotypic state at WBSCR17 site 2 (cfa6.7), genotypic state at GTF2I (cfa6.66), genotypic state at POM121 (cfa6.83), Breed, Age, and Sex.

Table S2. Significant loops with chr6:5.752-6.754Mb, containing the polymorphic TE site at *GTF2I*

Table S3. Normalized Exon and Junction expression of *GTF2I*

Table S4. Protein-coding genes in the differentially expressed module with respect to TE genotypic state

Table S5. *ChIP* TF enrichment analyses for 147 protein coding genes in the differentially expressed gene module

Table S6. IP reactions with Anti E2F1 and anti-H3K27ac antibodies.

Table S7. Capture C Library Statistics

Table S8. *ChIP*-Seq Library Statistics

Table S9. RNA-Seq Library Statistics

Table S10. Differentially Expressed Genes (FDR<0.1) for six samples with paired Capture C and RNA-Seq data

Supplementary References

**Materials and Methods**

**Sample acquisition:** Brainstem samples were collected from pet dogs, which had been euthanized for medical reasons by veterinarians at veterinary clinics. We only included dogs without major illnesses linked to the nervous system, which were also explored during the dissection. The dogs’ bodies were donated to the Canine Brain and Tissue Bank by their owners, with consent from the veterinarians who performed the euthanasia. Both owners and veterinarians had been informed about the goals of the donations, which were to collect and store biological specimens for scientific purposes. Sample collection and stabilization took place at the facility of the CBTB within a strict time frame of four hours following the euthanasia of animals. Brains were removed from the skulls in whole and were briefly rinsed in PBS. Next, tissue pieces from specific brain regions were either immersed in *RNAlater* (~100 mg tissue/1 ml solution) in 1.5 ml Eppendorf tubes or were fast frozen and placed in -80 °C ultra-low temperature freezers for long term storage. *RNAlater* stabilized tissue pieces were kept at 4 °C overnight to allow optimal penetration of the stabilizing solution and were subsequently put to -80 °C following the removal of the supernatant. Transportation of samples between countries was done in dry ice supplemented packages to avoid the thawing of fast frozen samples.

**Determination of TE genotypic state at *WBSCR17*, *GTF2I* and *POM121*:** To genotype individuals for the ancestral and derived alleles across all three genes, we extracted DNA from paired buccal swabs or <10mg brain tissue preserved in *RNAlater* with Qiagen’s DNeasy Blood and Tissue Kit (Qiagen, Germantown, MD, USA), using the manufacturer protocol for the respective tissue type. For *RNAlater* tissue, we first rinsed the tissue with 1X PBS prior to following kit manufacturer recommendations for DNA extraction from tissue. We then used our previously published PCR protocol (*1*) to generate amplicons of approximately 250bp (lacking the TE insertion) and 500bp (containing the TE insertion) in length. We visualized the PCR products on a 1.8% TAE agarose gel for genotyping.

***Capture C* bait design:** We used the CanFam3.1 genome assembly (GCF_000002285.3) to design 120-mer capture baits. Due to the repetitive nature of the TE insertion sites, we designed baits to capture sites that flanked the TE allele with 1Kb of sequence both upstream and downstream using the Agilent Sure Design resource. We designed additionally 120-mer baits to target the putative promoters of all genes within our original target region (CFA6:2,031,491-7,215,670 base pairs). We used the *liftOver* (*2*) tool functionality of the UCSC genome browser to identify putative promoters using experimental data from the hg19 human genome. Subsequently, we used *BLAT* to validate the absence of non-specific bait binding against the dog CanFam3.1 and CanFam4 genome assemblies to ensure binding specificity at the target regions despite the possibility of breed-specific sequence differences.

***DNAse Capture* C library preparation:** We prepared *DNase* HiC libraries using the Dovetail Omni C kit and 20mg of flash-frozen dog brainstem tissue. We ground tissue to fine powder in liquid nitrogen and subsequently resuspended it in 1x PBS. Thereafter, we used 10ul of 0.1M DSG and 27ul of 37% formaldehyde for sample crosslinking. We digested the crosslinked DNA using 3ul Dovetail non-specific DNA endonuclease. We opted for random digestions as they substantially reduce the likelihood of identifying false positives by stringent PCR duplicate removal. We performed ligations and prepared the library as per kit manufacturer’s recommendations. We used Dovetail dual indexes to prevent tagmentation, amplified the *DNAse* HiC libraries using 12 PCR cycles, and concentrated libraries with 3x AMPure XP beads (Beckman Coulter). We used 300ng of each HiC library for subsequent pull down, capture, and final amplification using manufacturer recommendations of the Agilent SureSelect XT HS kit and 14 PCR cycles. We then submitted the libraries for paired-end sequencing (2x150nt) at the Princeton University Lewis Sigler’s Genomics core facility on either a *MiSEQ* or spiked into a Novaseq 6000 Illumina platform. Our goal was to obtain at least 225 reads mapping to the polymorphic *GTF2I* insertion site for downstream analyses.

***Capture C* library alignment and quality control:** We first trimmed raw FASTQ files using *Trimgalore* v0.0.6 (*3*) to remove low quality reads (*q*<20) that were subsequently aligned to the CanFam3.1 reference genome (GCF_000002285.3) using the Burrows-Wheel Aligner (*bwa-mem* v0.7.17-r1188) on the ‘-5SP’ mode (*4*). We used *pairtools* v0.3.0 (*5*) with the *parse* command to organize sequence alignments into HiC pairs with the parameter settings *min-mapq*=40, *walks-policy*=*5unique*, and *max-iter-align-gap*=30. After sorting the pairs, we used *pairtools dedup* to remove PCR duplicates defined as reads that mapped to the same base pair coordinates on both ends. The likelihood of generating true HiC contacts along the same co-ordinates by random DNA digestion, as opposed to PCR duplication, remains low. We subsequently sorted and indexed the processed BAM file using *samtools* v1.9 (*6*).

***Capture C* analyses and loop annotation:** We used the bioinformatic tool *CHiCAGO* v1.22.0 (*7*) to annotate significant chromatin loops through the comparison of the observed contact frequency in each genomic bin against the expected frequency, which decays with increasing distance from the site of interest (*8*). Since our protocol involved random digestion of DNA using DNase, we binned our genome into non-overlapping bins 20Kb long (e.g., bin1=chrN:0-20Kb, bin2=chrN:20-40Kb, bin3=40-60Kb etc.). We left all *CHiCAGO* settings at default values except we used *minNPerBait*=50 solely for model parameter optimization due to a low number of target regions and read depth at baits targeting promoter regions. We only used baited regions around the polymorphic *GTF2I* TE locus for samples that had at least 500 reads to interpret biologically meaningful results. *CHiCAGO* is unable to incorporate overlapping bins in its sliding window analyses, with reads discarded that are located outside of such bins. To reduce the likelihood of false negatives, we repeated the analysis with staggered non-overlapping 20Kb bins located 10Kb from bins in our first analysis described above (e.g., bin1=chrN:0-10Kb, bin2=chrN:1-30Kb, bin3=30-50Kb, etc.). These bins were created using an in-silico HindIII digestion for which the *CHiCAGO* default parameters are most optimized (*9*). To identify 3D contacts unique to the TE insertion copy number state at *GTF2I*, we annotated loops separately for each genotypic state (i.e., heterozygous for ancestral insertion allele at *GTF2I* and homozygous for the no-insertion derived allele at *GTF2I*). We defined significance as loops with the lowest 0.5-percentile of *q* values for each analysis and had at least one sequence read confirming the loop for each of the two genotypic states.

**Immunoprecipitation**: To evaluate whether the three-dimensional contacts overlap with other regulatory signatures, we performed chromatin immunoprecipitation (*ChIP-Seq*) for the transcription factor *E2F1* and the *H3K27ac* histone acetylation marks. We extracted nuclei from flash frozen brainstem tissue using the Active Motif High sensitivity kit (Active Motif). Briefly, we re-suspended 50 mg of flash-frozen tissue in Tissue fixation solution (4.35 ml Molecular grade water, 0.5 ml Active Motif 10X PBS, and 140 ul 37% (v/v) formaldehyde). We rotated the tube for 8 minutes, added 51.5 ul of Active Motif Stop Solution, and followed by 5 minutes of rotation. We then homogenized the tissue using a pestle tube with 30 strokes on ice and spun the cells down at 4° C and 1250 g for 3 minutes. We washed the pellet twice with cold 1 ml PBS Wash Buffer (21.25 ml molecular grade water, 2.5 ml Active Motif 10X PBS, and 1.25 ml Active Motif Detergent). We then resuspended the cell pellet in 1ml Active Motif chromatin prep buffer supplemented with 1ul Active Motif protease inhibitor cocktail (PIC) and 1 ul Active Motif 100 mM phenylmethylsulfonyl fluoride (PMSF). We transferred the solution to a chilled Active Motif 1 ml Dounce homogenizer, incubated the solution on ice for 10 minutes, and then released nuclei with 50 strokes using the tight-fitting pestle Type A. We transferred the homogenate to a new 1.5 ml tube, spun the solution down for 3 minutes at 1250 g and at 4° C, and resuspended the solution in 750 ul of Active Motif Chromatin Prep Buffer supplemented with 7.5 ul Active Motif PIC and 7.5 ul Active Motif 100 mM PMSF. We sonicated the sample on a Branson 450 Sonifier in a cold room and on ice (output control=2; Duty Cycle=Constant) with 20 pulses of 10 second sonication ‘on’ duration and 50 seconds resting on ice. To remove cellular debris, we spun the tube for 2 minutes at 4° C and maximum speed, transferred 25 ul of sonicated chromatin for Input DNA preparation and stored the remainder of the sonicated sample for downstream immunoprecipitation (IP). We extracted input DNA with the Active motif Chromatin IP DNA purification kit following the manufacturer’s recommendations. We quantitated the extracted DNA on a Qubit v1.0 and stored this extracted DNA for library preparation as a non-IP’ed negative control. We conducted IP using the Polyclonal anti-*E2F1* antibody from Cell signaling technologies (cat# 3742S, Lot 8; concentration 48 ug/ml) and polyclonal Anti-Histone *H3K27ac* antibody form Active Motif (cat# 39034; concentration 1 ug/ul). Each sample underwent two IP reactions: one with the anti-*E2F1* antibody and one with anti-*H3K27ac* antibody. We added components to each IP reaction as summarized in Table S6.

We rotated IP reaction tubes overnight at 4° C and conducted all subsequent steps as per the manufacturer protocol of the Active Motif ChIP IT High Sensitivity Kit. We prepared libraries using the manufacturer protocol of Active Motif Next Gen DNA library Prep kit and Active Motif Next Gen Indexing kit with 15 PCR cycles. Active Motif indices contain unique molecular identifiers at the i5 position, which we annealed to the DNA prior to PCR amplification for accurate PCR duplication removal during bio-informatic processing. In addition, we prepared libraries for the Input DNA that did not undergo any IP steps as negative controls. We collected 2x50nt paired end sequence data on a NovaSeq6000 at the Princeton University Genomics Core Facility.

***ChIP-Seq* analyses and peak discovery:** We performed *ChIP-Seq* deduplication and peak discovery to identify regions in the genome bound to *E2F1* and *H3K27ac*. We incorporated unique molecular identifiers into the sequencing read names using *umi_tools* v1.1.2 (*10*) with the extract functionality. We subsequently demultiplexed and removed low-quality reads using *Trimgalore* v0.0.6 (*3*), with *-q* set to 20. We then aligned sequence data using *bowtie* v2.2 (*11*) to the Canfam3.1 reference genome (GCF_000002285.3) under default parameters, used *samtools v1.9* (*6*) to sort reads by position. We removed PCR duplicates, as defined by reads that contained the same unique molecular identifiers and mapped to identical genomic coordinates, using the *umi_tools* with the *dedup* and *--unique* flag functionalities. Upon alignment with *bowtie2*, we default to keeping multi-mapped reads (*11*). However, for any given reads mapping to the genomic same position and with the same UMI barcode, the *umi_tools* *dedup* functionality preferentially keeps reads with unique alignments (*10*). Therefore, while we kept multi-mapped reads during the alignment step, while removing PCR duplicates, we kept the read with the unique alignment over the ‘duplictae’ with multiple alignments *(10)*. We annotated peaks individually for each sample using *macs2* v2.2.0 (*12*).

To compensate for a higher than usual observable noise for E2F1 ChIP-Seq tracks, we conducted stringent peak discovery with parameters that yielded peaks matching visual observations of each replicate track on Integrative Genome Viewer (IGV). Therefore, the conservative parameters for the macs2 *callpeak* functionally were *q=*5x10^-4^, *-f BAMPE and -g 2500000000*. For H2K27ac, we identified broad peaks using the following parameter settings for macs2 *callpeak*: *-f BAMPE, -g 2500000000, -m 5 50, --broad-cutoff 0.1,* and *--broad*. We identified regions with FDR<5x10^-4^ and FDR<0.05 as significant peaks for *E2F1* and *H3K27ac*, respectively. To look for differences in binding enrichment at peaks, especially around regions overlapping with three-dimensional contacts we identified, we conducted *DiffBind* *DESEQ2* analyses (*13-17*) for *E2F1* and *H3K27ac* using default parameters. *DiffBind* incorporates signal and noise contributed by all samples within the same condition. Due to varying sample-specific IP efficiency, as reflected by differences in background reads, the same peak may not be present or annotated in all samples within the same treatment group. *DiffBind* alleviates this problem to some extent by performing differential binding analyses at genomic regions that (in our case) are identified as peaks in at least two of the three samples within the same treatment group. It then compares the number of normalized reads at these selected regions between treatment groups. We also removed all peak sites that showed enrichment in the non-IP Input DNA, which were our negative controls, with the *dba.blacklist* function. While we were specifically interested in differential peaks occurring at the three-dimensional contact bins, we conducted genome-wide differential binding enrichment analyses and with FDR corrections to allow for accurate normalization and scan for signatures across the genome Due to an overly conservative and right-skewed *p* value distribution (Fig. S3), we subsequently applied FDR corrections using the R package *fdrtools* (*18,19*) to compute *p_adj_* more suited to the distribution of our test statistics. We considered adjusted *p*<0.1 as significant.

For peak visualization, we normalized all libraries to the largest library size and constructed smoothened *bedgraph* files with bin size set to 100, using *deepTools* v3.5.1 (*20*). We used *wiggletools* v1.2 (*21*) with the mean functionality to output *bedgraph* files with mean values across bins for all samples in the same treatment group to construct one representative plot for each treatment.

***RNA-Seq* library preparation:** We used 50mg of pons brainstem tissue preserved in *RNAlater* from 22 domestic dogs, six of which were also included in the *Capture C* methods (Table S1). We rinsed the tissue in cold 1X PBS and subsequently homogenized them in Zymo DNA/RNA Shield buffer in a Qiagen Tissue Lyser. We extracted RNA using Zymo’s RNA MiniPrep Plus kit following the manufacturer’s recommendations and further concentrated using RNAse-free 2X AMPure XP beads. We used the RNA integrity number (RIN>6.5) to select samples for library preparation with Zymo’s Ribo Deplete kit. We targeted total RNA for sequencing, as opposed to messenger RNA, to avoid data loss due to sample degradation. We submitted each sample for pair-end sequencing (2x150 nucleotide) on a NovaSeq6000 S1 flowcell to obtain approximately 50 million reads per sample.

**Quality control and alignment:** We demultiplexed raw reads using a custom python script (<https://github.com/JaneliaSciComp/msg/blob/master/barcode_splitter.py>) followed by *Trimgalore* v0.0.6 (*3*) to remove low quality reads (cutoff *Q*>20) and trim remnant adaptor sequences. We then aligned sequences to the Canfam3.1 reference genome (GCF_000002285.3) using *STAR* v2.7.6 (*22*) with *sjdbOverhang*=149. We used the *HTSeq* python script to compute gene counts with the parameters --*type*=genes and --*stranded*=reverse. We normalized read count data based on gene GC content and sample read depth, then conducted variance stabilization in the R package *EDAseq* v2.32.0 (*23*). We removed batch effects introduced by using different reagent batches using the *removeBatchEffect* correction on R package *limma* v3.34.1 (*24*).

**Differential local gene expression analyses:** Our next aim was to investigate local gene expression changes consistent with altered three-dimensional contacts. We filtered normalized gene count values to keep protein coding genes within the highest and lowest three-dimensional contact sites (in nucleotides) for any given TE genotype. Further, we implemented a Shapiro Wilk’s test for normality to keep all genes satisfying the normality assumption for downstream *ANOVA* analyses. We conducted linear regression *ANOVAs* on remaining genes using R’s *anova(lm())* function in the *stats* v4.2.1 package (*25*). For the six individuals with *Capture C* data, we included the sample age and *GTF2I* TE genotypic state as fixed effects. Since our six samples come from mixed-breed dogs with high genetic and phenotypic variation as observed in natural populations, we conducted a linear regression *ANOVA* for all samples with *RNA-Seq* data to increase power with sample age, sex and *GTF2I* TE genotypic state as fixed effects. We applied Bonferroni corrections using R’s *p.adjust* function with package *stats* (*25*). We conducted a higher-powered *ANOVA* analysis, using the same parameters and function, using all 22 dogs with *RNA-Seq* data for genes that were approaching significance (*p<*0.1) in the lower powered analysis with 6 dogs.

**Differential Exon and Junction Usage Analyses:** To test our hypothesis for intra-gene looping and alternative splicing at *GTF2I*, we conducted differential exon and junction usage analyses. Due to the postulated developmental stage specific expression of TFII-I isoforms (*26, 27*), we performed a simplistic alternative splicing analysis for our controlled sample set that included dogs with *Capture C* and IP data, which were already controlled for age and sex. We first prepared our aligned data for R package *JunctionSeq* v1.16.0 (*28*) input by using the *QoRTs* v1.3.6 (*29*) package with the QC function and the *--stranded* flag. We imported the data into *JunctionSeq* and used the *runJunctionSeq* command with *analysis.type* set to *junctionsAndExons* and *use.novel.junctions* set to *TRUE*. For this analysis, we included the entire genome to ensure accurate normalization of exon count values. *JunctionSeq* corrects exon and junction expression values to the overall gene expression (*28*).

**Global changes in gene regulation:** Changes to local gene expression can impact global gene expression through the downstream impacts of the local gene products. Sine gene module construction requires at least 12 samples (*30*), we focused on the 22 samples with *RNAseq* data for this section of the analyses. We identified significant gene modules as those that had *p*<0.05 with respect to TE genotypic state at *GTF2I*. We also looked for differentially expressed genes using *EDAseq* v2.32.0 (*23*) normalized gene count values, computed under default parameters. We completed differential gene expression analyses on *DESeq2* v1.38.3 (*31*) with TE genotypic state at *GTF2I* as our dependent variable and included additional cofounders (design=~Batch+Age+Sex+*GTF2I* TE state). We computed the Wald’s p statistic for *GTF2I* TE insertion versus no insertion state for all 22 samples with *RNA-Seq*. We identified genes with FDR<0.1 or genes with *p*<0.001 and log2Fold changes greater than a magnitude of 2 as differentially expressed. To help identify affected biological pathways, we conducted a gene module enrichment analysis. We used normalized count values from *EDASeq* (*23*) and removed the effects of confounders such as Batch, Sex and Age using the *empiricalBayesLM* function on *WGCNA* (*30, 32*), with default parameters. We then determined our soft-thresholding power using the *pickSoftThreshold* function with *corFunc* set to *cor*. We defined the optimal power as the value where scale free R^2^ leveled off with increasing power and reached 0.9. To generate signed gene modules, which are collections of genes with positively correlated expression levels, we implemented *blockwiseModules* to with *power* set to the appropriate power value, *corType=pearson, TOMType=signed, minModuleSize=50*, and *mergeCutHeight*=0.25. We calculated the Pearson Correlation co-efficient to quantify gene module enrichment with respect to the TE genotypic state at GTF2I, followed by estimation of the student asymptotic *p*-value for correlation using the *corPvalueStudent* function equipped within *WGCNA*. Significance values were then subjected to FDR corrections using the *p.adjust* function on R. For correlated modules with *p*<0.05, we additionally conducted two-tailed Welch Two Sample t-test using the *t.test* function on R to estimate differences in means of module eigengenes, which represent dimensionality reduced gene expression values from all genes in the module (*30, 32*). We performed a gene Ontology enrichment analysis with *g:Profiler* v e110_eg57_p18_4b54a898 (*33*) using gene symbols, default settings and the *organism* set to *Canis lupus familiars*. To identify common regulators of genes in the module that show significantly differential enrichment as a function of TE genotypic state at *GTF2I*, we additionally performed a transcription-factor enrichment analyses for the gene list in the module using the *ChIP-X Enrichment Analysis* web tool v3 (*34*) and reported transcription factors with the lowest 0.5 percentile mean rank scores (all with FDR<0.05), where a lower rank is indicative of greater significance.

Given the interaction of TFII-I with some histone deacetylases and *E2F* transcription factor family, we leveraged our *ChIP-Seq* data to additionally look for differences in peaks across the full genome as well as overall global enrichment of *H3K27ac* and *E2F1* marks. To evaluate if the composition of peak enrichment is altered at motifs for specific transcription factors, we conducted a motif enrichment analysis of FASTA sequences located within differential *H3K27ac and E2F1* peaks (*p_adj_*<0.1) on the web interface for *CentriMo* available under the web tool *M*ultiple *E*xpectation maximizations for *M*otif *E*licitation (MEME) (*35*) with default parameters. This was completed in comparison to the JASPAR2022 CORE vertebrates non-redundant v2 for the following sites: 1) sites with higher peak enrichment in samples with the derived TE lacking state of *GTF2I* (i.e. log Fold Change_[Derived/Ancestral]_>0; *p_adj_*<0.1); and 2) sites with higher peak enrichment in samples with the ancestral TE containing state of *GTF2I* (i.e., log Fold Change_[Derived/Ancestral]_<0; *p_adj_*<0.1). We additionally annotated differential *H3K27ac* and *E2F1* peaks using the *annotatePeaks.pl* script from Homer v4.11 (*36*) with the *Canis lupus familiaris* Ensembl GTF annotation file v102. We created spike in normalization factors for evaluating global changes in *E2F1* and *H3K27ac* enrichment following Egan et al. (2016) (*37*). Briefly, each IP reaction was spiked in with *Drosophila melanogaster* chromatin and H2Av antibody, which is a histone variant specific to *Drosophila*. We counted de-duplicated reads aligned to the *Drosophila* genome for six IP reactions conducted for *H3K27ac* and *E2F1* each. We computed normalization factors for each antibody IP set separately by dividing the number of unique *Drosophila* reads from each sample by the smallest number of unique *Drosophila* reads in the given antibody set. We then multiplied the total number of unique *Canis* reads with the corresponding normalization factor to obtain the final number of spike-in normalized reads. We conducted a Wilcoxon rank sum exact test using the *wilcox.test* function in R package *stats* (*25*) to get a statistical estimate for differences in global *H3K27ac* and *E2F1* enrichment. The *GTF2I* TE genotypic state was the independent variable, with spike-in normalized count values as the response variable and *p*<0.05 as the significance threshold.

**Supplementary Text**

**Library Statistics:** As part of our quality control, we have computed and reported library statistics of all generated libraries. These statistics are available in Tables S7-S9. We additionally validated *E2F1* *ChIP* binding profiles which were centered around the transcription start sites (Fig. S4), as past studies have shown that *E2F1* mostly binds to the transcription start sites and gene promoters *(38)*.

**Controls:** Appropriately preserved dog brain samples for the assays were difficult to acquire during our study period. We were able to control for breeds to some extent in six samples with flash frozen tissue that had paired *Capture C*, *ChIP-Seq* and *RNA-Seq* data- five dogs were mixed breed and one was a Labrador Retriever. All six samples were from males between the ages of 12 and 16 years. 22 samples with *RNA-Seq* data were a mixture of ages, sexes and breeds. We were able to validate a lack of a pronounced breed effect via a principal component analysis of *RNA-Seq* count values on *DESeq2 (31)*. We first transformed and normalized the count values on a log2 scale using the *vsd* function with *DESeq2* and then computed and plotted the principal components from these transformed values using the *plotPCA* function. We observed weak clustering with respect to sex and age, where females have higher PC1 scores, and older dogs have lower PC2 scores (Fig. S5A and S5D); we found no strong effects of batch and breed (Figs. S5B,C). We incorporated sex and age as additional fixed effects in our *DESeq2* design and removed the effects of these confounders using the *empiricalBayesLM* function on *WGCNA* (SI Appendix, Methods). Since we found no straightforward way to account for the age and sex variable in the differential exon analyses, which are carried out with respect to the overall expression of the gene, we limited that analysis to the six dogs with *Capture C* and *RNAseq* data.

**Selection of E2F1 for *ChIP-Seq*:** We identified binding motifs in the TE sequence through a web-based bio-informatic tool known as Patch available through the website gene-regulation.com (*39*), with the TE sequence as the input (canfam3.1 CFA6:5753797-5753983) and the following default parameters: sites search for=vertebrates; maximum number of mismatches=2; mismatch penalty=100; lower score boundary=87.5. The program predicts transcription factor binding sites in the input sequences using positional weight matrices available through TRANSFAC® Public 6.0 *(40)*. Among the binding motifs identified in the TE sequence (n=169), a fraction of the hits is for binding motifs for *E2F* proteins (n=11), *E2F1* co-factors such as *Sp1* (n=16) and *CEBP* proteins (n=3). We also scanned *E2F1* *ChIP-Seq* ENCODE datasets *(41-44)*, to confirm significant *ChIP* peaks (*p_adj_<0.05*) that are conserved across replicates (Irreproducible Discovery Rate (IDR) rescue and self-consistency ratios <2) located within 1Mb of *GTF2I* start and end coordinates.

**Supplemental analysis of gene expression:** To ensure the applicability of our *Capture C* and *ChIP-Seq* findings with global gene expression and pathway analyses with all 22 samples, we additionally implemented a differential gene expression analysis for six samples containing paired IP and *Capture C* data. We performed this analysis on *DESeq2* v1.38.3 *(31)* with TE genotypic state at *GTF2I* as our dependent variable (design=~ *GTF2I* TE state) and reported all genes with FDR<0.1 (Table S10). We also perform a gene-ontology enrichment analysis with *g:Profiler* *(33)* using default settings and the *organism* set to *Canis lupus familiars* for all differentially expressed genes. Genes such as *ADAMTS15* and *SLC14A1* are classified as differentially expressed in analyses containing all 22 samples and the analyses with 6 samples. In addition, for the differentially expressed genes among the 6 samples, we also get gene ontology terms related to the extra-cellular matrix with the top ranked and only term for the KEGG pathway “Cell Adhesion” (*p_adj_*=1.83x10^-2^). Interactions with the extra-cellular matrix components are facilitated by molecules involved in cell adhesion *(45)*.

**Other Chromatin Loops: Ancestral TE insertion possibly associated with higher expression of *LAT2***

The second altered 3D contact with a concordant differential *ChIP* peak signal was at *LIMK1*. We found unique contacts at 6.28-6.32Mb on CFA6 (log q<-10) in the presence of at least one copy of the ancestral *GTF2I* allele (Fig. S1A; Table S2). No such contacts were found in the presence of at least copy of the derived *GTF2I* allele (Fig. S1B; Table S2). We immunoprecipitated the active regulatory marker histone lysine 27 acetylation (*H3K27ac*) and found that samples with the ancestral allele had a modestly higher *ChIP* peak signal for *H3K27ac* within the gene *LIMK1* at 6,282,462-6,282,862 (log Fold Change _[Derived/Ancestral]_=-0.872, *p=*0.02, *p_adj_*=1.0) (Figs. S1A-C). While analyzing paired *RNA-seq* data from the 6 samples, we found a non-significant increase in the expression of *LAT2* (*p=*0.07), ecompassed within the *GTF2I-LIMK1* loop, when dogs were heterozygous for the TE insertion. With a higher-powered analysis of 22 dogs with *RNA-seq* data, we found *LAT2* to be differentially expressed (log2FC _[Derived/Ancestral]=_-0.513, *p=*0.012; *p_adj_*=0.13; Fig. S1D). We postulate that this chromatin loop could indirectly bring the *LAT2* enhancer proximal to its gene promoter, thereby driving its differential expression. However, since many pieces of evidence did not survive multiple comparison corrections at the genome-wide level, this postulation is suggestive and should be confirmed with higher-powered analyses and investigations of looping interactions at the *LAT2* promoter.

**Supplementary Figures**


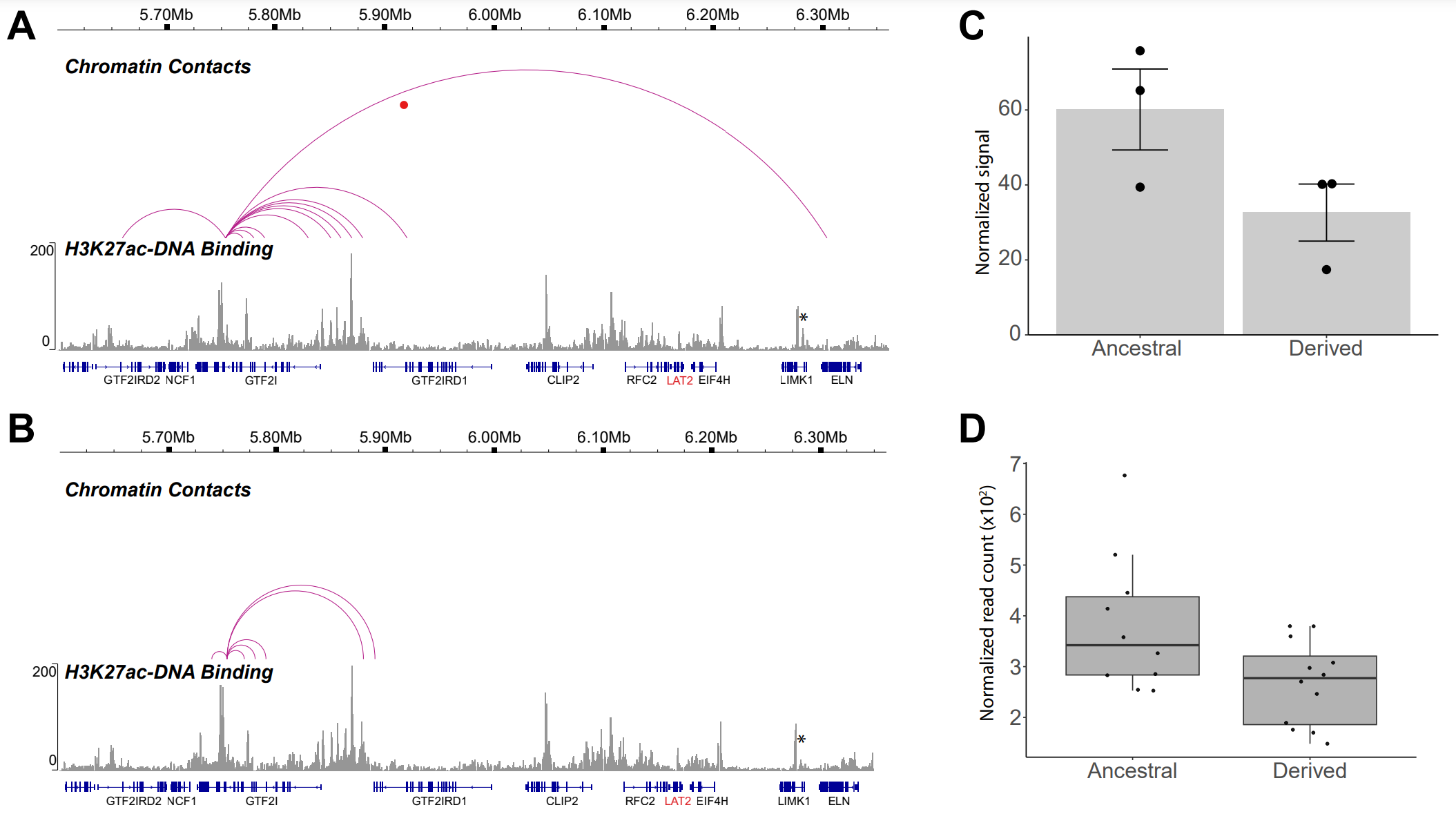


**Fig. S1.** **Putative differences in *cis-* regulation of *LAT2*, between ancestral TE present and derived TE absent states of *GTF2I*.** Visualization of the target region with chromosomal coordinates in Mb (top line) along canine chromosome CFA6 for *Capture C* contacts (top) and average *H3K27ac* *ChIP-Seq* Coverage (middle) with its differential peak (*) **A)** heterozygous for the ancestral TE insertion in *GTF2I* and **B)** homozygous for the derived allele lacking the TE insertion in *GTF2I*. For panels **C-D**, the ancestral state refers to the TE insertion in *GTF2I* while the derived allele is the lack of the TE insertion. **C)** Normalized *ChIP-Seq* signal for *H3K27ac* located at *LIMK1* (6,282,462-6,282,862bp, *p=*0.03, *p_adj_*=1.0). Black circles show each data point, bar heights correspond to group means and error bars correspond to standard errors. **D)** *LAT2* expression for all 22 samples containing *RNA-Seq* data (log2FC_[Derived/Ancestral]_=-0.513, *p*=0.012; *p_adj_*=0.13). Whiskers represent lower and upper data limits excluding outliers. Box edges correspond to 25 and 75 percentile ranges, and the horizontal line within the box represents the median value. Black circles show each data point. For simplicity and clarity, long-noncoding RNAs have been removed from the track.


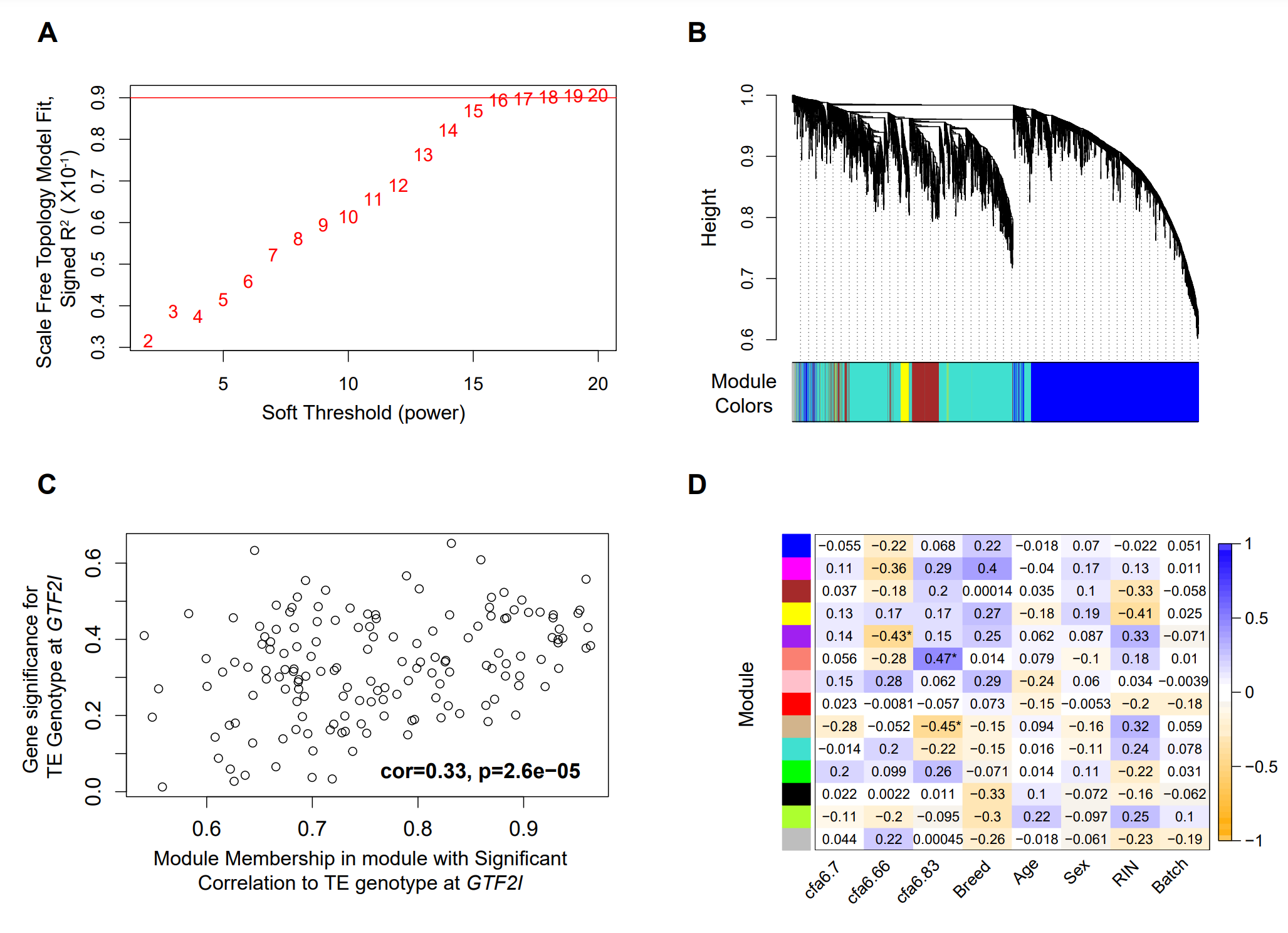


**Fig. S2.** **Construction of gene modules on WGCNA.** (A) Scatterplot used to determine the soft-thresholding “power” parameter (x-axis) for gene network construction, where the scale free topology (y-axis) levels off and meets 0.9 (at the horizontal red line); (B) Cluster dendrogram showing the hierarchical relationships between genes, used to assess their module membership (represented as different colors) by WGCNA. Not all module colors are visible, owning to relatively few genes within these modules (~50). Hence these appear as thin lines under the dendrogram, and are not visible at the current zoom resolution; (C) Scatterplot showing the association between a quantitative estimate of gene membership in the differentially expressed module (y-axis) and TE genotypic state (x-axis); (D) Heatmap displaying modules (represented as different colors; with the same color scheme and similar order [top to bottom] as those in panel B [left-right]) and their association, as quantified by the Pearson Correlation Coefficient, with the explanatory variables of TE genotypic state at *WBCR17* (cfa6.7), *GTF2I* (cfa6.66) , *POM121* (cfa6.83), breed, age, sex and batch. Cells marked with an Asterix (*) represent differentially expressed modules (*p*<0.05) for the corresponding explanatory variable.

**
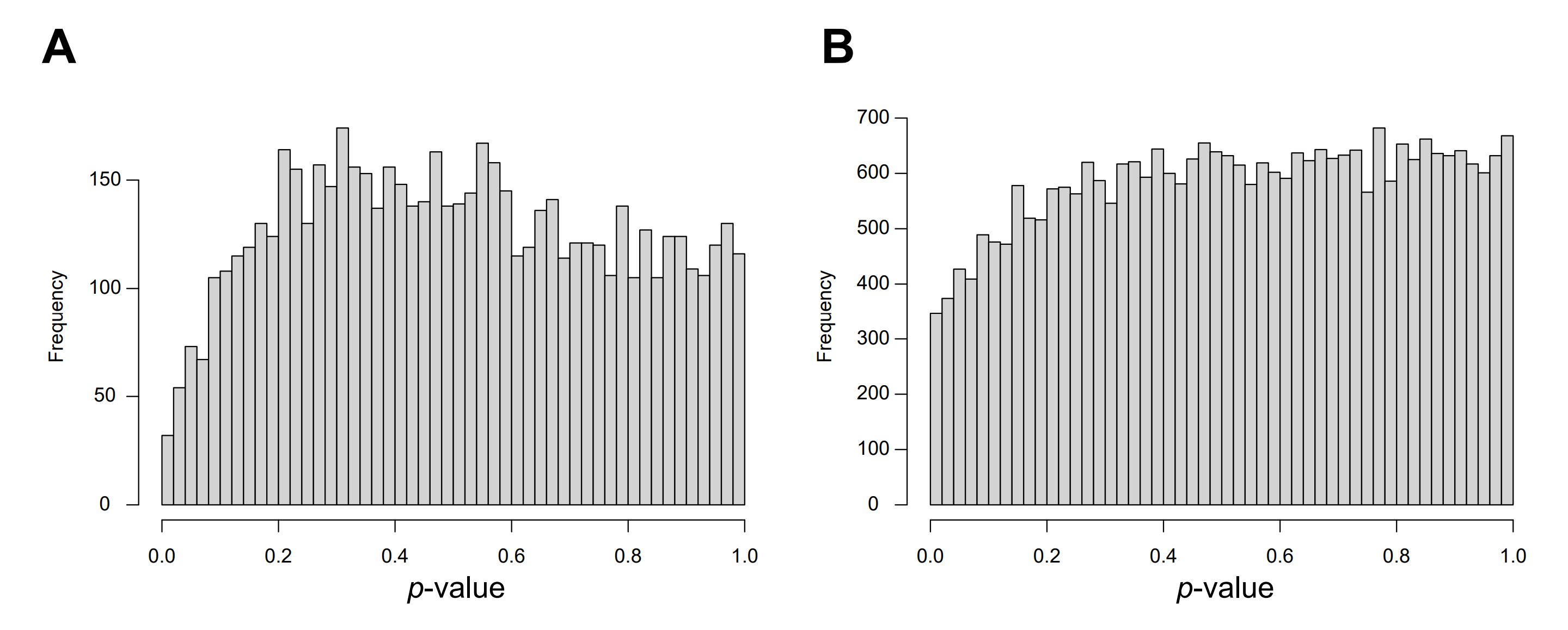
**

**Fig S3: Multiple comparisons adjustment for conservative *p*-values distributions.** *p*-value distribution output from differential *ChIP* peak enrichment analysis (DiffBind) for (A) anti-E2F1 and (B) anti-H3K27ac immunoprecipitation. Conservative *p* value distribution from these outputs led us to consider *fdrtools* for multiple comparison adjustments (*18,19*).


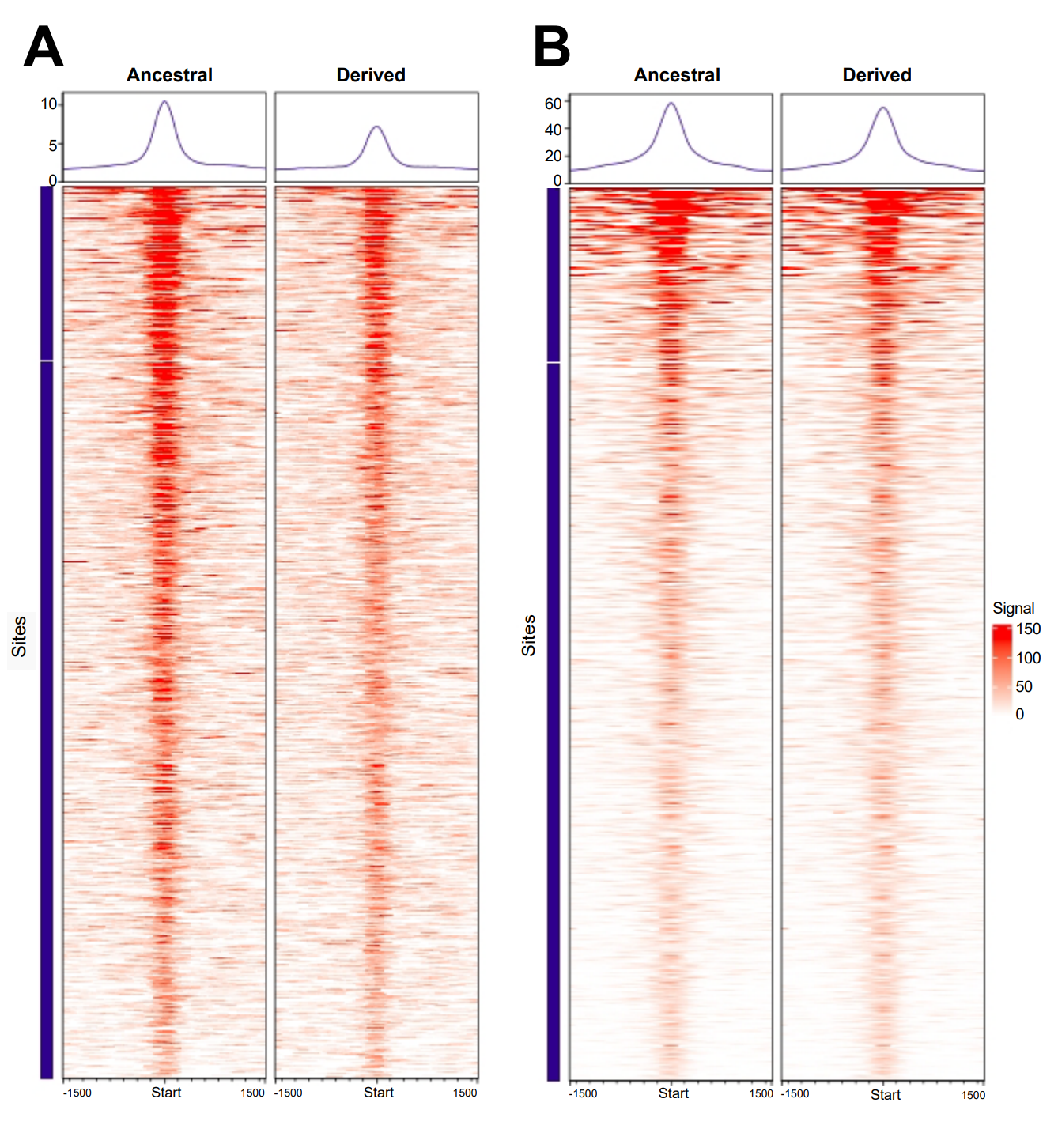


**Fig. S4. *ChIP-Seq* binding profile for (A) *E2F1* and (B) *H3K27ac*.** Line-plots in the top panel depict the enrichment of binding peaks (y axis) relative to the distance from the transcription start site (x axis). Enrichment profiles in the lower panels also depict protein binding relative to the transcription start sites (x axis), but at each site along the genome (x axis); deeper red is indicative of higher *ChIP-Seq* signal.


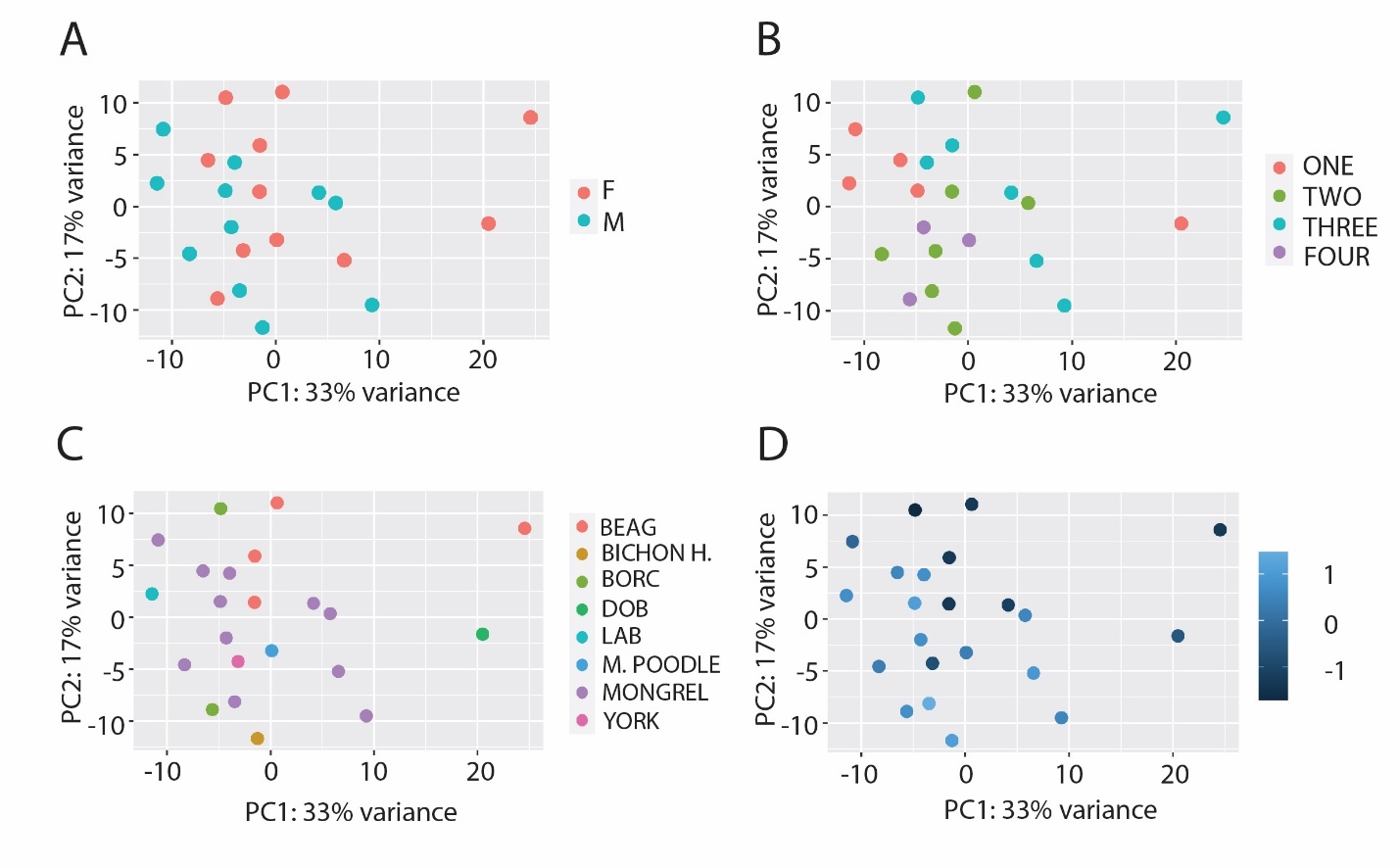


**Fig. S5. Principal component loadings of all 22 samples based on *RNA-Seq* count values.** Points are colored by sample (A) Sex (F=female; M=male); (B) Reagent Batch (assigned numbers); (C) Breed (BEAG=Beagle, BICHON H=Bichon Havanese, BORC=Border Collie, DOB=Doberman Pinscher, LAB=Labrador Retriever, M. POODLE=Miniature Poodle, MONGREL=Mixed-breed, YORK=Yorkshire Terrier); (D) Age scaled to range [1,-1], where lighter blue signifies older individuals.

**Supplementary Tables**

**Table S1.** Sample Metadata with Sample ID, genotypic state at *WBSCR17* site 1 (cha6.6), genotypic state at *WBSCR17* site 2 (cfa6.7), genotypic state at *GTF2I* (cfa6.66), genotypic state at *POM121* (cfa6.83), Breed, Age, and Sex. Genotypic numbers specify number of derived alleles where 0=homozygous for the ancestral allele, 1=heterozygous for, 2=homozygous for the derived allele, and NA=not amplified or failed PCR. The “Flash Frozen Tissue” column identifies samples with paired *RNALater* preserved and flash-frozen tissues (Y) and samples with only *RNALater* later preserved tissue (blank).

| Sample ID | Cfa6.6 | Cfa6.7 | Cfa6.66 | Cfa6.83 | Breed | Age | Sex | Flash Frozen Tissue |
| --- | --- | --- | --- | --- | --- | --- | --- | --- |
| 12517 | 1 | 0 | 1 | 1 | Labrador Retriever | 14 | Male | Y |
| 12518 | 1 | 1 | 2 | 0 | Mixed | 13 | Female |  |
| 12519 | 1 | 1 | 1 | 0 | Mixed | 15 | Female |  |
| 12521 | 2 | 2 | 1 | 0 | Beagle | 3 | Female |  |
| 12522 | 2 | 2 | 1 | 0 | Beagle | 3 | Female |  |
| 12523 | PCR Failure | 0 | 1 | 1 | Bichon Havanese | 16 | Male |  |
| 12527 | 2 | 1 | 1 | 2 | Mixed | 13 | Male | Y |
| 12528 | 2 | 2 | 2 | 0 | Border Collie | 1 | Female |  |
| 12533 | 2 | 2 | 2 | 2 | Border Collie | 14 | Female |  |
| 12534 | 1 | 1 | 1 | 0 | Dobermann | 7 | Female |  |
| 12537 | 1 | 1 | 2 | 1 | Mixed | 9 | Male |  |
| 12540 | 1 | 1 | 2 | 2 | Beagle | 3 | Female |  |
| 12544 | 2 | 1 | 2 | 1 | Beagle | 3 | Female |  |
| 12545 | 2 | 1 | 1 | 0 | Yorkshire Terrier | 6 | Female |  |
| 12546 | 1 | 0 | 1 | 1 | Mixed | 4 | Male |  |
| 12551 | 2 | 0 | 2 | 0 | Miniature Poodle | 12 | Female |  |
| 12552 | 2 | 2 | 2 | 0 | Mixed | 13 | Male | Y |
| 12554 | 1 | 0 | 2 | 0 | Mixed | 12 | Male | Y |
| 12556 | 1 | 0 | 2 | 0 | Mixed | 14 | Male |  |
| 12558 | 1 | 0 | 2 | 1 | Mixed | 14 | Male | Y |
| 12563 | 1 | 1 | 2 | 0 | Mixed | 18 | Male |  |
| 12568 | 1 | 0 | 1 | 0 | Mixed | 16 | Male | Y |

**Table S2.** Significant loops with chr6:5.752-6.754Mb (CanFam 3.1), containing the polymorphic TE site at *GTF2I*

| Ancestral allele containing the TE insertion | | | | | | | |
| --- | --- | --- | --- | --- | --- | --- | --- |
| Chr | Start (in bp) | Stop (in bp) | Replicate 1 raw read count | Replicate 2 raw read count | Replicate 3 raw read count | Mean raw read count | Log *q* (from its respective CHiCAGO analysis) |
| chr6 | 5650000 | 5670000 | 5 | 2 | 4 | 3.67 | -10.8292 |
| chr6 | 5760000 | 5780000 | 4 | 6 | 5 | 5.00 | -10.1389 |
| chr6 | 5770000 | 5790000 | 7 | 9 | 3 | 6.33 | -12.1266 |
| chr6 | 5780000 | 5800000 | 6 | 10 | 2 | 6.00 | -11.4722 |
| chr6 | 5820000 | 5840000 | 7 | 5 | 4 | 5.33 | -11.4068 |
| chr6 | 5841142 | 5860000 | 2 | 4 | 2 | 2.67 | -9.98934 |
| chr6 | 5850000 | 5870000 | 1 | 3 | 5 | 3.00 | -10.0459 |
| chr6 | 5859985 | 5878130 | 3 | 4 | 6 | 4.33 | -15.2804 |
| chr6 | 5860000 | 5880000 | 3 | 5 | 6 | 4.67 | -11.6592 |
| chr6 | 5870000 | 5890000 | 2 | 4 | 2 | 2.67 | -10.0997 |
| chr6 | 5910000 | 5930000 | 3 | 5 | 0 | 2.67 | -10.1702 |
| chr6 | 6289184 | 6320034 | 3 | 1 | 4 | 2.67 | -13.1394 |
| Derived allele lacking the TE insertion | | | | | | | |
| Chr | Start (in bp) | Stop (in bp) | Replicate 1 raw read count | Replicate 2 raw read count | Replicate 3 raw read count | Mean read count | Log *q* (from its respective CHiCAGO analysis) |
| chr6 | 5730000 | 5750000 | 10 | 6 | 2 | 6.00 | -10.9250 |
| chr6 | 5760000 | 5780000 | 13 | 2 | 4 | 6.33 | -10.8667 |
| chr6 | 5770000 | 5790000 | 9 | 3 | 3 | 5.00 | -10.5171 |
| chr6 | 5780000 | 5800000 | 7 | 1 | 5 | 4.33 | -10.0676 |
| chr6 | 5870000 | 5890000 | 7 | 3 | 0 | 3.33 | -10.8316 |
| chr6 | 5878131 | 5886169 | 3 | 4 | 0 | 2.33 | -12.4253 |
| chr6 | 5880000 | 5900000 | 5 | 3 | 1 | 3.00 | -9.97802 |

**Table S3: Normalized Exon and Junction expression of *GTF2I*:** Normalized counts of all annotated (Ensembl v102) exons and junctions of *GTF2I*. While CFA6 co-ordinates (CanFam 3.1) represent bin loci, junction coordinates merely represent end and start sites of the corresponding exons. Junction counts are quantified using reads spanning these loci and skipping alignment in the intronic regions, mimicking a discordant read with a deletion. *One unannotated junction was identified between exons 26 and 27 (i.e. Novel Exon 26-27)

| **Bin** | **CFA6**  **Start** | **CFA6**  **End** | ***Ancestral*** | | | ***Derived*** | | |
| --- | --- | --- | --- | --- | --- | --- | --- | --- |
|  |  |  | **12517** | **12527** | **12568** | **12552** | **12554** | **12558** |
| *Exons* |  |  |  |  |  |  |  |  |
| Exon1 | 5727664 | 5727797 | 329 | 521.1 | 340 | 335.2 | 195 | 433.9 |
| Exon2 | 5728507 | 5728583 | 412.8 | 297.9 | 435 | 431.4 | 258.8 | 538.8 |
| Exon3 | 5729094 | 5729136 | 400.5 | 296 | 370.7 | 394.2 | 215.8 | 404.1 |
| Exon4 | 5729925 | 5729967 | 497.6 | 403.9 | 432.2 | 488.8 | 266.9 | 448.7 |
| Exon5 | 5731374 | 5731403 | 500.6 | 403.9 | 452.7 | 510.6 | 283.2 | 506.3 |
| Exon6 | 5733027 | 5733211 | 723.3 | 845.6 | 774.9 | 879.9 | 427.8 | 803.6 |
| Exon7 | 5733573 | 5733657 | 503.7 | 673.4 | 614.7 | 654.9 | 307.7 | 629.9 |
| Exon8 | 5734097 | 5734178 | 355.5 | 425.6 | 507.6 | 431.4 | 217.3 | 445.9 |
| Exon9 | 5734586 | 5734645 | 262.6 | 275.2 | 364.2 | 350.7 | 148.3 | 384.6 |
| Exon10 | 5735989 | 5736173 | 411.7 | 407.6 | 496.4 | 558.7 | 213.5 | 441.3 |
| Exon11 | 5736522 | 5736588 | 265.6 | 235.5 | 298 | 338.3 | 126.8 | 232.3 |
| Exon12 | 5737122 | 5737224 | 319.8 | 248.7 | 475 | 322.8 | 149 | 310.3 |
| Exon13 | 5744277 | 5744352 | 346.3 | 341.4 | 449.9 | 422.1 | 200.2 | 409.7 |
| Exon14 | 5744745 | 5744804 | 318.8 | 385.9 | 430.3 | 451.6 | 215 | 450.6 |
| Exon15 | 5745428 | 5745612 | 454.6 | 475.7 | 554.2 | 571.1 | 289.9 | 566.7 |
| Exon16 | 5746666 | 5746738 | 403.6 | 325.4 | 415.4 | 369.4 | 221 | 375.3 |
| Exon17 | 5751592 | 5751651 | 423 | 457.8 | 482.5 | 355.4 | 224.7 | 406.9 |
| Exon18 | 5752803 | 5752987 | 680.4 | 770.8 | 761.9 | 505.9 | 284.7 | 611.3 |
| Exon19 | 5760177 | 5760249 | 588.5 | 576 | 815.9 | 488.8 | 283.2 | 569.5 |
| Exon20 | 5760824 | 5760883 | 550.7 | 634.6 | 781.4 | 518.3 | 296.6 | 602 |
| Exon21 | 5764131 | 5764315 | 697.8 | 824.7 | 1012.4 | 653.3 | 402.6 | 774.8 |
| Exon22 | 5767518 | 5767584 | 433.2 | 410.5 | 651 | 375.6 | 268.4 | 528.6 |
| Exon23 | 5769015 | 5769126 | 571.1 | 355.6 | 818.7 | 408.1 | 210.6 | 547.2 |
| Exon24 | 5776479 | 5776542 | 242.1 | 148.5 | 263.6 | 184.7 | 79.3 | 189.5 |
| Exon25 | 5778487 | 5778544 | 418.9 | 373.6 | 542.1 | 366.2 | 194.3 | 377.2 |
| Exon26 | 5780586 | 5780646 | 242.1 | 307.4 | 340 | 262.3 | 162.4 | 293.6 |
| Exon27 | 5789933 | 5790011 | 386.2 | 416.2 | 476.9 | 380.2 | 218 | 404.1 |
| Exon28 | 5799332 | 5799376 | 376 | 330.1 | 428.4 | 325.9 | 174.2 | 363.2 |
| Exon29 | 5799857 | 5799912 | 492.4 | 433.2 | 531.8 | 473.3 | 257.3 | 565.8 |
| Exon30 | 5805302 | 5805331 | 352.5 | 316.8 | 352.1 | 299.5 | 169.8 | 395.8 |
| Exon31 | 5805504 | 5805688 | 645.7 | 640.3 | 736.7 | 640.9 | 352.2 | 650.3 |
| Exon32 | 5806748 | 5806883 | 652.8 | 619.5 | 840.1 | 681.3 | 380.4 | 697.7 |
| Exon33 | 5809908 | 5810047 | 537.4 | 597.7 | 693 | 541.6 | 303.3 | 580.6 |
| Exon34 | 5811861 | 5811965 | 355.5 | 393.5 | 392.1 | 313.5 | 195.8 | 417.1 |
| *Junctions* |  |  |  |  |  |  |  |  |
| Exon1-Exon2 | 5727797 | 5728507 | 151.2 | 113.5 | 182.6 | 124.2 | 92.7 | 222 |
| Exon2-Exon3 | 5728583 | 5729094 | 338.2 | 210.9 | 326.9 | 324.3 | 192.8 | 360.5 |
| Exon3-Exon4 | 5729136 | 5729925 | 337.1 | 240.2 | 312 | 349.2 | 187.6 | 333.5 |
| Exon4-Exon5 | 5729967 | 5731374 | 457.7 | 354.7 | 394.9 | 433 | 238 | 414.3 |
| Exon5-Exon6 | 5731403 | 5733027 | 468.9 | 381.2 | 413.5 | 476.4 | 255.8 | 482.2 |
| Exon6-Exon7 | 5733211 | 5733573 | 379 | 552.4 | 465.7 | 484.2 | 229.1 | 442.2 |
| Exon7-Exon8 | 5733657 | 5734097 | 271.8 | 344.3 | 381.9 | 302.6 | 164.6 | 335.4 |
| Exon8-Exon9 | 5734178 | 5734586 | 234 | 234.6 | 314.8 | 293.3 | 128.3 | 333.5 |
| Exon9-Exon10 | 5734645 | 5735989 | 193.1 | 196.7 | 249.6 | 263.8 | 111.2 | 264.8 |
| Exon10-Exon11 | 5736173 | 5736522 | 224.8 | 216.6 | 260.8 | 290.2 | 99.4 | 193.2 |
| Exon11-Exon12 | 5736588 | 5737122 | 181.9 | 115.4 | 212.4 | 167.6 | 65.3 | 141.2 |
| Exon12-Exon13 | 5737224 | 5744277 | 197.2 | 140 | 304.6 | 184.7 | 94.2 | 195.1 |
| Exon13-Exon14 | 5744352 | 5744745 | 298.3 | 324.4 | 408 | 394.2 | 189.8 | 391.1 |
| Exon14-Exon15 | 5744804 | 5745428 | 253.4 | 322.5 | 310.2 | 374 | 166.8 | 364.2 |
| Exon15-Exon16 | 5745612 | 5746666 | 217.6 | 176.9 | 204.9 | 246.8 | 117.9 | 242.5 |
| Exon16-Exon17 | 5746738 | 5751592 | 310.6 | 262.9 | 318.5 | 254.5 | 151.3 | 271.3 |
| Exon17-Exon18 | 5751651 | 5752803 | 369.8 | 399.1 | 419.1 | 274.7 | 192 | 351.2 |
| Exon18-Exon19 | 5752987 | 5760177 | 384.1 | 371.7 | 415.4 | 214.2 | 100.8 | 299.1 |
| Exon19-Exon20 | 5760249 | 5760824 | 456.7 | 482.4 | 679.9 | 414.4 | 244.7 | 461.7 |
| Exon20-Exon21 | 5760883 | 5764131 | 452.6 | 539.1 | 703.2 | 454.7 | 271.4 | 513.8 |
| Exon21-Exon22 | 5764315 | 5767518 | 364.7 | 379.3 | 543 | 318.1 | 232.1 | 461.7 |
| Exon22-Exon23 | 5767584 | 5769015 | 343.3 | 260.1 | 529 | 240.5 | 164.6 | 398.6 |
| Exon23-Exon24 | 5769126 | 5776479 | 190 | 71.9 | 221.7 | 113.3 | 49.7 | 125.4 |
| Exon23-Exon25 | 5769126 | 5778487 | 82.8 | 31.2 | 122.9 | 35.7 | 18.5 | 49.2 |
| Exon24-Exon25 | 5776542 | 5778487 | 169.6 | 97.4 | 210.5 | 121 | 49.7 | 116.1 |
| Exon25-Exon26 | 5778544 | 5780586 | 174.7 | 199.6 | 251.5 | 183.1 | 91.9 | 166.3 |
| Exon25-Exon27 | 5778544 | 5789933 | 83.8 | 65.3 | 98.7 | 74.5 | 26 | 83.6 |
| Exon26-Exon27 | 5780646 | 5789933 | 173.7 | 194.8 | 243.1 | 159.8 | 92.7 | 180.2 |
| Exon27-Exon28 | 5790011 | 5799332 | 317.7 | 285.6 | 371.6 | 288.7 | 157.9 | 327 |
| Exon28-Exon29 | 5799376 | 5799857 | 307.5 | 263.9 | 350.2 | 253 | 146.8 | 306.6 |
| Exon29-Exon30 | 5799912 | 5805302 | 319.8 | 272.4 | 322.3 | 284 | 147.6 | 360.5 |
| Exon30-Exon31 | 5805331 | 5805504 | 314.7 | 290.4 | 318.5 | 279.3 | 160.2 | 360.5 |
| Exon31-Exon32 | 5805688 | 5806748 | 397.4 | 390.6 | 476.9 | 415.9 | 219.5 | 370.7 |
| Exon32-Exon33 | 5806883 | 5809908 | 345.3 | 312.1 | 518.8 | 341.4 | 198.7 | 384.6 |
| Exon33-Exon34 | 5810047 | 5811861 | 314.7 | 378.3 | 364.2 | 263.8 | 167.6 | 334.4 |
| Novel Exon26-27* | 5780643 | 5789933 | 29.6 | 54.9 | 31.7 | 38.8 | 16.3 | 45.5 |

**Table S4.** List of genes in the differentially expressed gene module (i.e. collection of co-expressed genes representing biological pathways) with respect to TE genotypic state

| Ensembl ID (v102) | Gene Symbol | Gene Description |
| --- | --- | --- |
| ENSCAFG00000006188 | *ADGRA2* | adhesion G protein-coupled receptor A2 |
| ENSCAFG00000002917 | *AEBP1* | AE binding protein 1 |
| ENSCAFG00000006861 | *ANGPT4* | angiopoietin 4 |
| ENSCAFG00000014005 | *ANGPTL1* | angiopoietin like 1 |
| ENSCAFG00000012013 | *ANPEP* | alanyl aminopeptidase, membrane |
| ENSCAFG00000003314 | *ANXA4* | annexin A4 |
| ENSCAFG00000002307 | *ASPN* | asporin |
| ENSCAFG00000010157 | *BACE2* | beta-secretase 2 |
| ENSCAFG00000019189 | *BGN* | biglycan |
| ENSCAFG00000014346 | *C1S* | complement C1s |
| ENSCAFG00000018608 | *C7* | complement C7 |
| ENSCAFG00000003169 | *CALD1* | caldesmon 1 |
| ENSCAFG00000007788 | *CAPG* | capping actin protein, gelsolin like |
| ENSCAFG00000020276 | *CCN1* | cellular communication network factor 1 |
| ENSCAFG00000029442 | *CCN2* | cellular communication network factor 2 |
| ENSCAFG00000030440 | *CCN3* | cellular communication network factor 3 |
| ENSCAFG00000016447 | *CD5L* | CD5 molecule like |
| ENSCAFG00000012919 | *CDC25A* | cell division cycle 25A |
| ENSCAFG00000032274 | *CINP* | cyclin dependent kinase 2 interacting protein |
| ENSCAFG00000005898 | *CKAP2* | cytoskeleton associated protein 2 |
| ENSCAFG00000014049 | *CLEC3B* | C-type lectin domain family 3 member B |
| ENSCAFG00000008404 | *CLU* | clusterin |
| ENSCAFG00000017018 | *COL1A1* | collagen type I alpha 1 chain |
| ENSCAFG00000002069 | *COL1A2* | collagen type I alpha 2 chain |
| ENSCAFG00000014812 | *COL3A1* | collagen type III alpha 1 chain |
| ENSCAFG00000011911 | *COL6A1* | collagen type VI alpha 1 chain |
| ENSCAFG00000012226 | *COL6A3* | collagen type VI alpha 3 chain |
| ENSCAFG00000014616 | *COMP* | cartilage oligomeric matrix protein |
| ENSCAFG00000017807 | *CRYM* | crystallin mu |
| ENSCAFG00000020404 | *CRYZ* | crystallin zeta |
| ENSCAFG00000010206 | *CTSE* | cathepsin E |
| ENSCAFG00000007026 | *CXCL12* | C-X-C motif chemokine ligand 12 |
| ENSCAFG00000032339 | *CYP2A13* | cytochrome P450 family 2 subfamily A polypeptide 13 |
| ENSCAFG00000005052 | *CYP2B6* | cytochrome P450 2B11 |
| ENSCAFG00000018622 | *DAB2* | DAB adaptor protein 2 |
| ENSCAFG00000016792 | *DCAF4* | DDB1 And CUL4 Associated Factor 4 |
| ENSCAFG00000006142 | *DCN* | decorin |
| ENSCAFG00000006359 | *DCUN1D2* | defective in cullin neddylation 1 domain containing 2 |
| ENSCAFG00000015475 | *DES* | desmin |
| ENSCAFG00000000803 | *DLA-DRA* | MHC class II DR alpha chain |
| ENSCAFG00000002098 | *ECRG4* | ECRG4 augurin precursor |
| ENSCAFG00000028947 | *ELN* | elastin |
| ENSCAFG00000007810 | *ERAP1* | endoplasmic reticulum aminopeptidase 1 |
| ENSCAFG00000007348 | *F11* | coagulation factor XI |
| ENSCAFG00000016161 | *FAM114A1* | family with sequence similarity 114 member A1 |
| ENSCAFG00000005598 | *FAM168A* | family with sequence similarity 168 member A |
| ENSCAFG00000016138 | *FBLIM1* | filamin binding LIM protein 1 |
| ENSCAFG00000000804 | *FBLN1* | fibulin 1 |
| ENSCAFG00000019521 | *FLNA* | filamin A |
| ENSCAFG00000017329 | *FLRT2* | fibronectin leucine rich transmembrane protein 2 |
| ENSCAFG00000014345 | *FN1* | fibronectin 1 |
| ENSCAFG00000019916 | *FOXC2* | forkhead box C2 |
| ENSCAFG00000014633 | *FRMD6* | FERM domain containing 6 |
| ENSCAFG00000024988 | *FRZB* | frizzled related protein |
| ENSCAFG00000001906 | *FZD1* | frizzled class receptor 1 |
| ENSCAFG00000014134 | *FZD2* | frizzled class receptor 2 |
| ENSCAFG00000010035 | *GABRA5* | gamma-aminobutyric acid type A receptor subunit alpha5 |
| ENSCAFG00000004579 | *GIMAP4* | GTPase, IMAP family member 4 |
| ENSCAFG00000029537 | *GJB2* | gap junction protein beta 2 |
| ENSCAFG00000005802 | *GPR183* | G protein-coupled receptor 183 |
| ENSCAFG00000013217 | *GPRC5A* | G protein-coupled receptor class C group 5 member A |
| ENSCAFG00000003663 | *GSN* | gelsolin |
| ENSCAFG00000010090 | *IGF2* | insulin like growth factor 2 |
| ENSCAFG00000014474 | *IGFBP5* | insulin like growth factor binding protein 5 |
| ENSCAFG00000012844 | *IL10RA* | interleukin 10 receptor subunit alpha |
| ENSCAFG00000017866 | *ISLR* | immunoglobulin superfamily containing leucine rich repeat |
| ENSCAFG00000011577 | *KCNJ13* | potassium inwardly rectifying channel subfamily J member 13 |
| ENSCAFG00000008896 | *KIAA1755* | KIAA1755 |
| ENSCAFG00000002797 | *KLF4* | Kruppel like factor 4 |
| ENSCAFG00000005037 | *KLF5* | Kruppel like factor 5 |
| ENSCAFG00000001106 | *LAMA2* | laminin subunit alpha 2 |
| ENSCAFG00000025057 | *LAMB1* | laminin subunit beta 1 |
| ENSCAFG00000011744 | *LAMB2* | laminin subunit beta 2 |
| ENSCAFG00000002964 | *LRRC4B* | leucine rich repeat containing 4B |
| ENSCAFG00000005133 | *LTBP4* | latent transforming growth factor beta binding protein 4 |
| ENSCAFG00000006138 | *LUM* | lumican |
| ENSCAFG00000007580 | *LYVE1* | lymphatic vessel endothelial hyaluronan receptor 1 |
| ENSCAFG00000000426 | *LYZ* | lysozyme |
| ENSCAFG00000015596 | *MAEL* | maelstrom spermatogenic transposon silencer |
| ENSCAFG00000009664 | *MATN4* | matrilin 4 |
| ENSCAFG00000012963 | *MGP* | matrix Gla protein |
| ENSCAFG00000000184 | *MOXD1* | monooxygenase DBH like 1 |
| ENSCAFG00000012806 | *MPZL2* | myelin protein zero like 2 |
| ENSCAFG00000004496 | *MRC1* | mannose receptor C-type 1 |
| ENSCAFG00000016934 | *NGFR* | nerve growth factor receptor |
| ENSCAFG00000008786 | *NNAT* | neuronatin |
| ENSCAFG00000010476 | *NOTCH2* | notch receptor 2 |
| ENSCAFG00000015893 | *NSUN7* | NOP2/Sun RNA methyltransferase family member 7 |
| ENSCAFG00000031564 | *NUDT4* | nudix hydrolase 4 |
| ENSCAFG00000002313 | *OGN* | osteoglycin |
| ENSCAFG00000011940 | *PARP9* | poly(ADP-ribose) polymerase family member 9 |
| ENSCAFG00000014322 | *PCOLCE* | procollagen C-endopeptidase enhancer |
| ENSCAFG00000012472 | *PDE5A* | phosphodiesterase 5A |
| ENSCAFG00000013778 | *PLA2G4A* | phospholipase A2 group IVA |
| ENSCAFG00000013691 | *PRG4* | proteoglycan 4 |
| ENSCAFG00000024193 | *PROB1* | proline rich basic protein 1 |
| ENSCAFG00000007945 | *PROCR* | protein C receptor |
| ENSCAFG00000029912 | *PRRT4* | proline rich transmembrane protein 4 |
| ENSCAFG00000007292 | *PSD4* | pleckstrin and Sec7 domain containing 4 |
| ENSCAFG00000018780 | *RAB34* | RAB34, member RAS oncogene family |
| ENSCAFG00000018711 | *RANBP3L* | RAN binding protein 3 like |
| ENSCAFG00000003620 | *RCN3* | reticulocalbin 3 |
| ENSCAFG00000010603 | *RGS1* | regulator of G protein signaling 1 |
| ENSCAFG00000009245 | *RHOBTB2* | Rho related BTB domain containing 2 |
| ENSCAFG00000000366 | *SASH1* | SAM and SH3 domain containing 1 |
| ENSCAFG00000015271 | *SERPIND1* | serpin family D member 1 |
| ENSCAFG00000024189 | *SERPINF1* | serpin family F member 1 |
| ENSCAFG00000020151 | *SF3B3* | splicing factor 3b subunit 3 |
| ENSCAFG00000029797 | *SIX1* | SIX homeobox 1 |
| ENSCAFG00000003306 | *SLC13A4* | solute carrier family 13 member 4 |
| ENSCAFG00000015972 | *SLC16A13* | solute carrier family 16 member 13 |
| ENSCAFG00000000757 | *SLC22A2* | solute carrier family 22 member 2 |
| ENSCAFG00000015418 | *SLC22A6* | solute carrier family 22 member 6 |
| ENSCAFG00000000212 | *SLC2A12* | solute carrier family 2 member 12 |
| ENSCAFG00000018195 | *SLC47A1* | solute carrier family 47 member 1 |
| ENSCAFG00000015051 | *SLC5A5* | solute carrier family 5 member 5 |
| ENSCAFG00000015756 | *SLC6A12* | solute carrier family 6 member 12 |
| ENSCAFG00000013947 | *SLC6A20* | solute carrier family 6 member 20 |
| ENSCAFG00000003749 | *SLC7A11* | solute carrier family 7 member 11 |
| ENSCAFG00000002692 | *STON1* | protein phosphatase 1 regulatory subunit 21 |
| ENSCAFG00000002692 | *STON1* | stonin 1 |
| ENSCAFG00000004006 | *SVIL* | supervillin |
| ENSCAFG00000015819 | *SYNE2* | spectrin repeat containing nuclear envelope protein 2 |
| ENSCAFG00000002987 | *TBX18* | T-box transcription factor 18 |
| ENSCAFG00000010832 | *TC2N* | tandem C2 Domains |
| ENSCAFG00000031894 | *TENT5A* | terminal nucleotidyltransferase 5A |
| ENSCAFG00000008975 | *THBS4* | thrombospondin 4 |
| ENSCAFG00000030694 | *TMEM38A* | transmembrane protein 38A |
| ENSCAFG00000015383 | *TNFSF10* | TNF superfamily member 10 |
| ENSCAFG00000009845 | *TTF2* | transcription termination factor 2 |
| ENSCAFG00000009323 | *UEVLD* | UEV and lactate/malate dehyrogenase domains |
| ENSCAFG00000023591 | *VNN1* | vanin 1 |
| ENSCAFG00000014354 | *VSIR* | V-set immunoregulatory receptor |
| ENSCAFG00000018672 | *VTN* | vitronectin |
| ENSCAFG00000003496 | *WNT16* | Wnt family member 16 |
| ENSCAFG00000014661 | *WNT4* | Wnt family member 4 |
| ENSCAFG00000005530 | *ZFP36* | ZFP36 ring finger protein |
| ENSCAFG00000020273 | *ZNHIT6* | zinc finger HIT-type containing 6 |
| ENSCAFG00000028494 | NA | Unidentified Protein Coding gene |
| ENSCAFG00000000641 | NA | Unidentified Protein Coding gene |
| ENSCAFG00000000806 | NA | Unidentified Protein Coding gene |
| ENSCAFG00000004242 | NA | Unidentified Protein Coding gene |
| ENSCAFG00000044133 | NA | Unidentified Protein Coding gene |
| ENSCAFG00000031869 | NA | Unidentified Protein Coding gene |
| ENSCAFG00000049474 | NA | Unidentified Protein Coding gene |
| ENSCAFG00000043492 | NA | Unidentified Protein Coding gene |
| ENSCAFG00000010290 | NA | Unidentified Protein Coding gene |

**Table S5.** *ChIP* transcription factor (TF) enrichment analyses for 147 protein coding genes in the differentially expressed gene module using the *ChIP*-X Enrichment Analysis web tool v3 (*34*). This analysis helps identify common regulators of co-expressed genes that make up a gene module (*30,32)*. Mean rank represents integrated ranks across all publicly available libraries listed in the “Library” Column, whereby lower ranks are indicative of greater significance and higher gene overlap. All TFs are ranked with FDR<0.05 in their respective library.

| Rank | TF | Mean Rank | Overlapping Genes | Library |
| --- | --- | --- | --- | --- |
| 1 | [*PRRX2*](https://maayanlab.cloud/Harmonizome/gene/PRRX2) | 3.0 | 44 | ARCHS4 Coexpression,7; Enrichr Queries,1; GTEx Coexpression,1 |
| 2 | [*TWIST2*](https://maayanlab.cloud/Harmonizome/gene/TWIST2) | 3.5 | 32 | ARCHS4 Coexpression,3; GTEx Coexpression,4 |
| 3 | [*TWIST1*](https://maayanlab.cloud/Harmonizome/gene/TWIST1) | 7.75 | 50 | ARCHS4 Coexpression,4; Enrichr Queries,4;ReMap *ChIP-Seq*,1;GTEx Coexpression,22 |
| 4 | [*OSR1*](https://maayanlab.cloud/Harmonizome/gene/OSR1) | 10.0 | 40 | ARCHS4 Coexpression,1; GTEx Coexpression,19 |
| 5 | [*BNC2*](https://maayanlab.cloud/Harmonizome/gene/BNC2) | 10.33 | 42 | ARCHS4 Coexpression,13; Enrichr Queries,9;GTEx Coexpression,9 |
| 6 | [*FOXS1*](https://maayanlab.cloud/Harmonizome/gene/FOXS1) | 12.67 | 38 | ARCHS4 Coexpression,22; Enrichr Queries,12;GTEx Coexpression,4 |
| 7 | [*AEBP1*](https://maayanlab.cloud/Harmonizome/gene/AEBP1) | 13.0 | 34 | ARCHS4 Coexpression,13; GTEx Coexpression,13 |
| 8 | [*HEYL*](https://maayanlab.cloud/Harmonizome/gene/HEYL) | 13.33 | 39 | ARCHS4 Coexpression,26; Enrichr Queries,2;GTEx Coexpression,12 |
| 9 | [*PRRX1*](https://maayanlab.cloud/Harmonizome/gene/PRRX1) | 14.0 | 37 | ARCHS4 Coexpression,2; Enrichr Queries,10;GTEx Coexpression,30 |

**Table S6.** IP reactions with Anti *E2F1* and anti-*H3K27ac* antibodies.

| *Anti-E2F1 IP* | |
| --- | --- |
| Component | **Volume added (in ul)** |
| Sonicated Chromatin | volume containing 500ng |
| Active Motif PIC | 5 |
| Antibody Blocker Mix  *[10ul anti-E2F1 antibody+5ul Active Motif Blocker]* | 15 |
| Active Motif Spike in Chromatin (1ng/ul) | 0.4 |
| Active Motif Spike in Antibody | 2 |
| *ChIP* Buffer | Remainder amount to bring up to 240ul |
| *Anti H3K27ac IP* | |
| Component | **Volume Added (in ul)** |
| Sonicated Chromatin | volume containing 500ng |
| Active Motif PIC | 5 |
| Antibody Blocker Mix  *[4ul anti-H3K27ac antibody+5ul Active Motif Blocker]* | 9 |
| Active Motif Spike in Chromatin (1ng/ul) | 1 |
| Active Motif Spike in Antibody | 2 |
| *ChIP* Buffer | Remainder amount to bring up to 240ul |

**Table S7.** *Capture C* Library Statistics

| Sample ID | Total Reads  Pairs | Aligned Read Pairs | PCR No-Dup Read Pairs | Cis Read Pairs | Trans Read Pairs | Unique Reads Mapping to *GTF2I* Bait (chr6:5752533-5755065) |
| --- | --- | --- | --- | --- | --- | --- |
| 12517 | 3,039,600 | 2,714,146 | 500,006 | 423,465 | 76,541 | 578 |
| 12527 | 2,657,689 | 2,348,763 | 486,684 | 360,966 | 125,718 | 734 |
| 12552 | 2,041,608 | 1,848,565 | 499,903 | 432,632 | 67,271 | 840 |
| 12554 | 2,646,524 | 2,348,938 | 700,931 | 536,104 | 164,827 | 602 |
| 12558 | 5,333,883 | 4,749,507 | 536,000 | 446,742 | 89,258 | 541 |
| 12568 | 812,254 | 406,480 | 296,995 | 164,285 | 132,710 | 756 |

**Table S8.** *ChIP-Seq* Library Statistics. (Abbreviations: FragL=Fragment Length; RelCC=Relative strand cross-correlation coefficient; SSD=Standard Deviation of Signal Pile-up; RiP%=Percentage of reads within discovered macs2 peaks; NA=Not Applicable)

| Sample ID | Antibody | Total Reads | Total Mapped Reads | Paired and  Mapped  Reads | Unique Read Pairs | Frag.L | Rel.CC | SSD | RiP% |
| --- | --- | --- | --- | --- | --- | --- | --- | --- | --- |
| 12517 | *E2F1* | 11207114 | 59947139 | 56329484 | 9663083 | 225 | 1.11 | 14.5 | 13 |
| 12517 | H3K27ac | 126493324 | 116497213 | 106362878 | 27201745 | 277 | 7.74 | 7.52 | 60.4 |
| 12517 | Input Control | 117652760 | 114135324 | 106583398 | 35703545 | 239 | 5.81 | 0.798 | NA |
| 12527 | *E2F1* | 92922122 | 74724392 | 70696208 | 10422165 | 219 | 0.99 | 15.1 | 14 |
| 12527 | H3K27ac | 118651912 | 112729100 | 100880396 | 30310806 | 293 | 8.05 | 10.3 | 62.7 |
| 12527 | Input Control | 101765430 | 98823759 | 91574154 | 27435743 | 240 | 7.65 | 0.542 | NA |
| 12552 | *E2F1* | 114990142 | 79733156 | 75863820 | 14555258 | 222 | 1.33 | 27.2 | 15.6 |
| 12552 | H3K27ac | 110048268 | 103917212 | 90913060 | 30512655 | 292 | 8.24 | 14 | 59.5 |
| 12552 | Input Control | 99722002 | 94575409 | 89391020 | 28516628 | 251 | 6.21 | 0.768 | NA |
| 12554 | *E2F1* | 96040714 | 81373124 | 76823320 | 16383452 | 224 | 1.39 | 24.3 | 14.3 |
| 12554 | H3K27ac | 131198346 | 123648365 | 109118918 | 34334861 | 288 | 9.06 | 3.51 | 68.9 |
| 12554 | Input Control | 116623872 | 112691369 | 105402752 | 33785918 | 243 | 6.79 | 0.67 | NA |
| 12558 | *E2F1* | 85959894 | 54508225 | 52577610 | 8066322 | 214 | 0.95 | 18.2 | 14.6 |
| 12558 | H3K27ac | 118275450 | 111977661 | 104263502 | 25349234 | 288 | 8.03 | 1.64 | 62.5 |
| 12558 | Input Control | 94797408 | 92414851 | 85194392 | 27769008 | 252 | 5.96 | 0.678 | NA |
| 12568 | *E2F1* | 105560488 | 63187082 | 60485582 | 8858446 | 217 | 1.13 | 21.6 | 17.9 |
| 12568 | H3K27ac | 140417344 | 129228814 | 117958852 | 33236586 | 276 | 8.67 | 4.55 | 67.2 |
| 12568 | Input Control | 127278788 | 123128494 | 115644626 | 35417457 | 237 | 7.03 | 0.709 | NA |

**Table S9.** *RNA-Seq* Library Statistics

| Sample ID | Number of input reads | Average input read length | Uniquely mapped reads number | RIN | Batch |
| --- | --- | --- | --- | --- | --- |
| 12517 | 64321836 | 300 | 55956104 | 7.2 | One |
| 12518 | 83253545 | 300 | 75815829 | 7.5 | One |
| 12519 | 80083175 | 300 | 71489854 | 6.7 | Four |
| 12521 | 77923608 | 293 | 62268735 | 7.0 | Two |
| 12522 | 66220308 | 300 | 60959309 | 6.9 | Four |
| 12523 | 73895520 | 297 | 63022296 | 7.7 | Two |
| 12527 | 75210191 | 299 | 67675362 | 7.5 | Four |
| 12528 | 52668541 | 300 | 48191799 | 7.5 | Four |
| 12533 | 48813180 | 290 | 38070487 | 7.5 | Three |
| 12534 | 70085055 | 299 | 60437459 | 7.4 | One |
| 12537 | 84221638 | 300 | 75935355 | 7.8 | One |
| 12540 | 58185528 | 297 | 50004459 | 6.6 | Two |
| 12544 | 50004459 | 299 | 46181542 | 7.0 | Four |
| 12545 | 65035153 | 297 | 55797913 | 6.9 | Two |
| 12546 | 66537546 | 300 | 60465474 | 7.4 | Four |
| 12551 | 50641707 | 292 | 41413105 | 7.6 | Three |
| 12552 | 48266109 | 297 | 40185017 | 7.1 | Two |
| 12554 | 85486825 | 294 | 71280886 | 7.6 | Two |
| 12556 | 60158325 | 300 | 55325180 | 7.3 | Four |
| 12558 | 73784539 | 296 | 62815566 | 6.6 | Three |
| 12563 | 73476276 | 297 | 62807540 | 7.5 | Two |
| 12568 | 68637155 | 300 | 63302215 | 7.6 | One |

**Table S10.** Differentially Expressed Genes (FDR<0.1) for six samples with paired *Capture C* and *RNA-Seq* data

| Ensembl ID (v102) | gene Symbol | Full Name | log2FC | p value | FDR |
| --- | --- | --- | --- | --- | --- |
| ENSCAFG00000009961 | *ADAMTS15* | ADAM metallopeptidase with thrombospondin type 1 motif 15 | -3.67918 | 1.47x10^-5^ | 1.76x10^-2^ |
| ENSCAFG00000028797 | *ADM5* | adrenomedullin 5 (putative) | 3.559991 | 2.53x10^-6^ | 5.63x10^-3^ |
| ENSCAFG00000003314 | *ANXA4* | annexin A4 | -1.15278 | 8.05x10^-5^ | 4.38x10^-2^ |
| ENSCAFG00000028936 | *ARMC12* | armadillo repeat containing 12 | -1.51537 | 1.25x10^-4^ | 5.56x10^-2^ |
| ENSCAFG00000024085 | *BLOC1S3* | biogenesis of lysosomal organelles complex 1 subunit 3 | 2.944877 | 2.88x10^-5^ | 2.49x10^-2^ |
| ENSCAFG00000017005 | *BORCS6* | BLOC-1 related complex subunit 6 | 1.776517 | 3.36x10^-5^ | 2.75x10^-2^ |
| ENSCAFG00000012729 | *CGN* | cingulin | 1.407636 | 1.55x10^-4^ | 6.37x10^-2^ |
| ENSCAFG00000042795 | *CLDN5* | claudin 5 | 1.787397 | 5.10x10^-6^ | 7.72x10^-3^ |
| ENSCAFG00000003710 | *CRYBG1* | crystallin beta-gamma domain containing 1 | -1.23897 | 1.36E-08 | 1.06x10^-4^ |
| ENSCAFG00000023386 | *CTXN1* | cortexin 1 | 2.473277 | 2.36x10^-4^ | 7.66x10^-2^ |
| ENSCAFG00000009366 | *DAPL1* | death associated protein like 1 | -1.71642 | 9.02x10^-5^ | 4.53x10^-2^ |
| ENSCAFG00000010260 | *DCDC2* | doublecortin domain containing 2 | -1.47137 | 7.00x10^-5^ | 4.04x10^-2^ |
| ENSCAFG00000032469 | *DDIT4* | DNA damage inducible transcript 4 | -1.387 | 5.45x10^-6^ | 7.72x10^-3^ |
| ENSCAFG00000017925 | *DLK1* | delta like non-canonical Notch ligand 1 | 3.90058 | 6.59x10^-5^ | 4.04x10^-2^ |
| ENSCAFG00000009570 | *DSP* | dentin sialophosphoprotein | -1.76538 | 2.41x10^-7^ | 1.25x10^-3^ |
| ENSCAFG00000017716 | *EEF2K* | eukaryotic elongation factor 2 kinase | -0.68563 | 1.64x10^-4^ | 6.56x10^-2^ |
| ENSCAFG00000009199 | *ENDOU* | endonuclease, poly(U) specific | 1.350843 | 1.30x10^-4^ | 5.64x10^-2^ |
| ENSCAFG00000016699 | *FGFRL1* | fibroblast growth factor receptor like 1 | 1.869211 | 1.83x10^-4^ | 6.96x10^-2^ |
| ENSCAFG00000019165 | *GABRQ* | gamma-aminobutyric acid type A receptor subunit theta | 3.347472 | 8.16x10^-5^ | 4.38x10^-2^ |
| ENSCAFG00000040996 | *H3C3* | H3 clustered histone 3 | 2.724437 | 4.61x10^-5^ | 3.12x10^-2^ |
| ENSCAFG00000032754 | *HKDC1* | hexokinase domain containing 1 | -1.62649 | 1.88x10^-4^ | 6.98x10^-2^ |
| ENSCAFG00000014995 | *LRRC74B* | leucine rich repeat containing 74B | -1.84972 | 8.80x10^-5^ | 4.53x10^-2^ |
| ENSCAFG00000006533 | *MMP17* | matrix metallopeptidase 17 | 1.727119 | 1.42x10^-4^ | 5.97x10^-2^ |
| ENSCAFG00000012964 | *MPZ* | myelin protein zero | -1.68687 | 4.07x10^-5^ | 3.02x10^-2^ |
| ENSCAFG00000012119 | *MYLK* | myosin light chain kinase | -1.14535 | 1.88x10^-5^ | 1.83x10^-2^ |
| ENSCAFG00000031953 | *OLIG1* | oligodendrocyte transcription factor 1 | 1.560122 | 1.88x10^-5^ | 1.83x10^-2^ |
| ENSCAFG00000029311 | *PCP4* | Purkinje cell protein 4 | -1.4876 | 2.75x10^-4^ | 8.75x10^-2^ |
| ENSCAFG00000006748 | *PDYN* | prodynorphin | 4.622079 | 4.52x10^-6^ | 7.72x10^-3^ |
| ENSCAFG00000007063 | *PENK* | proenkephalin | 1.662258 | 1.24x10^-5^ | 1.61x10^-2^ |
| ENSCAFG00000003144 | *RAMP3* | receptor activity modifying protein 3 | 2.786745 | 2.82x10^-5^ | 2.49x10^-2^ |
| ENSCAFG00000032569 | *RBP1* | AT-rich interaction domain 4A | -1.27435 | 9.55x10^-5^ | 4.65x10^-2^ |
| ENSCAFG00000014713 | *REX1BD* | required for excision 1-B domain containing | 1.669908 | 6.82x10^-5^ | 4.04x10^-4^ |
| ENSCAFG00000019130 | *RFLNB* | refilin B | -1.34277 | 3.00x10^-10^ | 4.67x10^-6^ |
| ENSCAFG00000013889 | *RTP4* | receptor transporter protein 4 | -1.55475 | 3.73x10^-5^ | 2.90x10^-2^ |
| ENSCAFG00000015169 | *SELE* | selectin E | 3.297597 | 1.79x10^-4^ | 6.96x10^-2^ |
| ENSCAFG00000017664 | *SLC14A1* | solute carrier family 14 member 1 (Kidd blood group) | -1.23896 | 5.80x10^-5^ | 3.76x10^-2^ |
| ENSCAFG00000019037 | *SLITRK4* | SLIT and NTRK like family member 4 | 1.706275 | 1.07x10^-4^ | 4.97x10^-2^ |
| ENSCAFG00000025327 | *SOX17* | SRY-box transcription factor 17 | 2.97465 | 4.58x10^-5^ | 3.12x10^-2^ |
| ENSCAFG00000013891 | *SST* | somatostatin | 5.406422 | 3.85x10^-7^ | 1.50x10^-3^ |
| ENSCAFG00000004032 | *TAL1* | TAL bHLH transcription factor 1, erythroid differentiation factor | 1.104962 | 1.97x10^-4^ | 7.03x10^-2^ |
| ENSCAFG00000029615 | *TCF24* | transcription factor 24 | 2.045131 | 1.66x10^-5^ | 1.83x10^-2^ |
| ENSCAFG00000028849 | *TMEM141* | transmembrane protein 141 | -1.06862 | 1.99x10^-4^ | 7.03x10^-2^ |
| ENSCAFG00000004588 | *TRH* | thyrotropin releasing hormone | 4.76181 | 2.33x10^-6^ | 5.63x10^-3^ |
| ENSCAFG00000000364 | *WIF1* | WNT inhibitory factor 1 | -1.94 | 2.19x10^-4^ | 7.41x10^-2^ |
| ENSCAFG00000015427 | *ZNF503* | zinc finger protein 503 | 2.186658 | 5.42x10^-6^ | 7.72x10^-3^ |
| ENSCAFG00000005851 | Unidentified Protein Coding gene | NA | 2.408813 | 5.68x10^-7^ | 1.77x10^-3^ |
| ENSCAFG00000014564 | Unidentified Protein Coding gene | NA | -1.45437 | 2.03x10^-4^ | 7.03x10^-2^ |
| ENSCAFG00000046356 | Unidentified Protein Coding gene | NA | 1.808379 | 2.30x10^-4^ | 7.61x10^-2^ |
| ENSCAFG00000046600 | Unidentified Protein Coding gene | NA | 1.41726 | 1.09x10^-4^ | 4.97x10^-2^ |

**Supplementary References**

1. Tandon D, Ressler K, Petticord D, Papa A, Jiranek J, Wilkinson R, et al. Homozygosity for Mobile Element Insertions Associated with WBSCR17 Could Predict Success in Assistance Dog Training Programs. Genes, 2019;10:439. 10.3390/genes10060439
2. Hinrichs AS. The UCSC Genome Browser Database: update 2006. Nucleic Acids Res. 2006;34:D590–8. [10.1093/nar/gkj144](https://academic.oup.com/nar/article-lookup/doi/10.1093/nar/gkj144)
3. Martin M. Cutadapt removes adapter sequences from high-throughput sequencing reads. EMBnet j. 2011;17(1):10. 10.14806/ej.17.1.200
4. Li H, Durbin R. Fast and accurate short read alignment with Burrows–Wheeler transform. Bioinformatics. 2009;25:1754–60. 10.1093/bioinformatics/btp324
5. Open2C, et al., “Pairtools: from sequencing data to chromosome contacts” (preprint, Bioinformatics, 2023), doi:[10.1101/2023.02.13.528389](https://doi.org/10.1101/2023.02.13.528389).
6. Danecek P, Bonfield JK, Liddle J, Marshall J, Ohan V, Pollard MO, et al. Twelve years of SAMtools and BCFtools. GigaScience. 2021;10(2):giab008. 10.1093/gigascience/giab008/6137722
7. Cairns J, Freire-Pritchett P, Wingett SW, Várnai C, Dimond A, Plagnol V, et al. CHiCAGO: robust detection of DNA looping interactions in Capture Hi-C data. Genome Biol. 2016;17:127. 10.1186/s13059-016-0992-2
8. Lajoie BR, Dekker J, Kaplan N. The Hitchhiker’s guide to Hi-C analysis: Practical guidelines. Methods. 2015;72:65–75. 10.1016/j.ymeth.2014.10.031
9. Freire-Pritchett P, Ray-Jones H, Della Rosa M, Eijsbouts CQ, Orchard WR, Wingett SW, et al. Detecting chromosomal interactions in Capture Hi-C data with CHiCAGO and companion tools. Nat Protoc. 2021;16:4144–76. 10.1038/s41596-021-00567-5
10. Smith T, Heger A, Sudbery I. UMI-tools: modeling sequencing errors in Unique Molecular Identifiers to improve quantification accuracy. Genome Res. 2017;27:491–9. 10.1101/gr.209601.116
11. Langmead B, Salzberg SL. Fast gapped-read alignment with Bowtie 2. Nat Methods. 2012;9:357–9. 10.1038/nmeth.1923
12. Zhang Y, Liu T, Meyer CA, Eeckhoute J, Johnson DS, Bernstein BE, et al. Model-based Analysis of *ChIP*-Seq (MACS). Genome Biol. 2008;9:R137. 10.1186/gb-2008-9-9-r137
13. Ross-Innes CS, Stark R, Teschendorff AE, Holmes KA, Ali HR, Dunning MJ, et al. Differential oestrogen receptor binding is associated with clinical outcome in breast cancer. Nature. 2012;481:389–93. 10.1038/nature10730
14. R. Stark, G. Brown, DiffBind: differential binding analysis of *ChIP*-Seq peak data. (2011) doi:[10.18129/B9.BIOC.DIFFBIND](https://doi.org/10.18129/B9.BIOC.DIFFBIND).
15. Robinson MD, McCarthy DJ, Smyth GK. edgeR : a Bioconductor package for differential expression analysis of digital gene expression data. Bioinformatics. 2010;26:139–40. 10.1093/bioinformatics/btp616
16. McCarthy DJ, Chen Y, Smyth GK. Differential expression analysis of multifactor RNA-Seq experiments with respect to biological variation. Nucleic Acids Res. 2012;40:4288–97. 10.1093/nar/gks042
17. Chen Y, Lun ATL, Smyth GK. From reads to genes to pathways: differential expression analysis of RNA-Seq experiments using Rsubread and the edgeR quasi-likelihood pipeline. F1000Res. 2016;5:1438. 10.12688/f1000research.8987.2
18. Strimmer K. A unified approach to false discovery rate estimation. BMC Bioinform. 2008;9:1-4. 10.1186/1471-2105-9-303
19. Strimmer K. fdrtool: a versatile R package for estimating local and tail area-based false discovery rates. Bioinform. 2008;24:1461-2. 10.1093/bioinformatics/btn209
20. Ramírez F, Ryan DP, Grüning B, Bhardwaj V, Kilpert F, Richter AS, et al. deepTools2: a next generation web server for deep-sequencing data analysis. Nucleic Acids Res. 2016;44:W160–5. 10.1093/nar/gkw257
21. Zerbino DR, Johnson N, Juettemann T, Wilder SP, Flicek P. WiggleTools: parallel processing of large collections of genome-wide datasets for visualization and statistical analysis. Bioinformatics. 2014;30:1008–9. 10.1093/bioinformatics/btt737
22. Dobin A, Davis CA, Schlesinger F, Drenkow J, Zaleski C, Jha S, et al. STAR: ultrafast universal RNA-seq aligner. Bioinformatics. 2013;29(1):15–21. 10.1093/bioinformatics/bts635
23. Risso D, Schwartz K, Sherlock G, Dudoit S. GC-Content Normalization for RNA-Seq Data. BMC Bioinform. 2011;12:480. [10.1186/1471-2105-12-480](https://bmcbioinformatics.biomedcentral.com/articles/10.1186/1471-2105-12-480)
24. Ritchie ME, Phipson B, Wu D, Hu Y, Law CW, Shi W, et al. limma powers differential expression analyses for RNA-sequencing and microarray studies. Nucleic Acids Res. 2015;43:e47–e47. 10.1093/nar/gkv007
25. R Core Team, R: A language and environment for statistical computing, version 4.2.1, R Foundation for Statistical Computing (2022). URL <https://www.R-project.org/>.
26. Tapial J, Ha KCH, Sterne-Weiler T, Gohr A, Braunschweig U, Hermoso-Pulido A, et al. An atlas of alternative splicing profiles and functional associations reveals new regulatory programs and genes that simultaneously express multiple major isoforms. Genome Res. 2017;27:1759–68. [10.1101/gr.220962.117](http://genome.cshlp.org/lookup/doi/10.1101/gr.220962.117)
27. Cheriyath V, Roy AL. Alternatively Spliced Isoforms of TFII-I. J Biol Chem. 2000;275:26300–8. 10.1074/jbc.M002980200
28. Hartley SW, Mullikin JC. Detection and visualization of differential splicing in RNA-Seq data with JunctionSeq. Nucleic Acids Res. 2016;gkw501. 10.1093/nar/gkw501
29. Hartley SW, Mullikin JC. QoRTs: a comprehensive toolset for quality control and data processing of RNA-Seq experiments. BMC Bioinform. 2015;16:224. [10.1186/s12859-015-0670-5](https://bmcbioinformatics.biomedcentral.com/articles/10.1186/s12859-015-0670-5)
30. Langfelder P, Horvath S. WGCNA: an R package for weighted correlation network analysis. BMC Bioinform. 2008;9:559. [10.1186/1471-2105-9-559](https://bmcbioinformatics.biomedcentral.com/articles/10.1186/1471-2105-9-559)
31. Love MI, Huber W, Anders S. Moderated estimation of fold change and dispersion for RNA-seq data with DESeq2. Genome Biol. 2014;15:550. [10.1186/s13059-014-0550-8](http://genomebiology.biomedcentral.com/articles/10.1186/s13059-014-0550-8)
32. Zhang B, Horvath S. A General Framework for Weighted Gene Co-Expression Network Analysis. Stat Appl Genet Mol Biol. 2005;4. [10.2202/1544-6115.1128/html](https://www.degruyter.com/document/doi/10.2202/1544-6115.1128/html)
33. Raudvere U, Kolberg L, Kuzmin I, Arak T, Adler P, Peterson H, et al. g:Profiler: a web server for functional enrichment analysis and conversions of gene lists (2019 update). Nucleic Acids Res. 2019;47:W191–8. 10.1093/nar/gkz369
34. Lachmann A, Xu H, Krishnan J, Berger SI, Mazloom AR, Ma’ayan A. ChEA: transcription factor regulation inferred from integrating genome-wide *ChIP*-X experiments. Bioinformatics. 2010;26:2438–44. 10.1093/bioinformatics/btq466
35. Bailey TL, Machanick P. Inferring direct DNA binding from *ChIP*-seq. Nucleic Acids Res. 2012;40:e128–e128. 10.1093/nar/gks433
36. Heinz S, Benner C, Spann N, Bertolino E, Lin YC, Laslo P, Cheng JX, Murre C, Singh H, Glass CK. Simple combinations of lineage-determining transcription factors prime cis-regulatory elements required for macrophage and B cell identities. Mol cell. 2010;38:576-89. 10.1016/j.molcel.2010.05.004
37. Egan B, Yuan CC, Craske ML, Labhart P, Guler GD, Arnott D, et al. An Alternative Approach to *ChIP-Seq* Normalization Enables Detection of Genome-Wide Changes in Histone H3 Lysine 27 Trimethylation upon EZH2 Inhibition. Qin Z, editor. PLoS ONE. 2016;11:e0166438. [10.1371/journal.pone.0166438](https://dx.plos.org/10.1371/journal.pone.0166438)
38. Bieda M, Xu X, Singer MA, Green R, Farnham PJ. Unbiased location analysis of E2F1-binding sites suggests a widespread role for E2F1 in the human genome. Genome Res. 2006;16:595–605. [10.1101/gr.4887606](http://genome.cshlp.org/lookup/doi/10.1101/gr.4887606)
39. Gene-regulation.com. <http://gene-regulation.com/>. Accessed 20 February 2022.
40. Matys V. TRANSFAC(R) and its module TRANSCompel(R): transcriptional gene regulation in eukaryotes. Nucleic Acids Res. 2006;34:D108–10. [10.1093/nar/gkj143](https://academic.oup.com/nar/article-lookup/doi/10.1093/nar/gkj143)
41. Zhang J, Liu J, Lee D, Lou S, Chen Z, Gürsoy G, Gerstein M. DiNeR: a Differential graphical model for analysis of co-regulation network rewiring. BMC Bioinform. 2020;21:1-5. 10.1186/s12859-020-03605-3
42. Zhang X-O, Gingeras TR, Weng Z, Data from “Genome-wide analysis of polymerase III–transcribed *Alu* elements suggests cell-type–specific enhancer function.” Encyclopedia of DNA Elements (ENCODE). Available at doi:10.17989/ENCSR563LLO Deposited June 30^th^ 2015.
43. Zhang J, et al. Data from, “An integrative ENCODE resource for cancer genomics” Encyclopedia of DNA Elements (ENCODE). Available at doi:10.17989/ENCSR000EVJ Deposited December 6^th^ 2010
44. Zhang J, et al., Data from, “An integrative ENCODE resource for cancer genomics” Encyclopedia of DNA Elements (ENCODE). Available at doi:10.17989/ENCSR000EWX Deposited December 6^th^ 2010
45. Sagvolden G, Giaever I, Pettersen EO, Feder J, Cell adhesion force microscopy. *Proc. Natl. Acad. Sci. U.S.A.* 1999;96:471–476. 10.1073/pnas.96.2.471
